# Socio-Environmental Risks Linked with Mine Tailings Chemical Composition: Promoting Responsible and Safe Mine Tailings Management Considering Copper and Gold Mining Experiences from Chile and Peru

**DOI:** 10.3390/toxics11050462

**Published:** 2023-05-16

**Authors:** Carlos Cacciuttolo, Deyvis Cano, María Custodio

**Affiliations:** 1 Civil Works and Geology Department, Catholic University of Temuco, Temuco 4780000, Chile; 2 Facultad de Ciencias Forestales y Conservación de la Naturaleza, Universidad de Chile, Santiago 8320000, Chile; 3 Facultad de Ingeniería, Universidad Peruana de Ciencias Aplicadas, Lima 15023, Peru; 4 Programa Académico de Ingeniería Ambiental, Universidad de Huánuco, Huánuco 10001, Peru; 5 Centro de Investigación de Medicina en Altura y Medio Ambiente, Facultad de Medicina Humana, Universidad Nacional del Centro del Perú, Av. Mariscal Castilla N° 3909, Huancayo 12006, Peru

**Keywords:** copper mine tailings, gold mine tailings, metal–metalloid components, non-metal components, metallurgical reagents, acid rock drainage, metal leaching potential, toxicity, risks, responsible tailings management

## Abstract

There is a need to define mine tailings in a clear, precise, multidisciplinary, transdisciplinary, and holistic manner, considering not only geotechnical and hydraulic concepts but also integrating environmental and geochemical aspects with implications for the sustainability of mining. This article corresponds to an independent study that answers questions concerning the definition of mine tailings and the socio-environmental risks linked with mine tailings chemical composition by examining the practical experience of industrial-scale copper and gold mining projects in Chile and Peru. Definitions of concepts and analysis of key aspects in the responsible management of mine tailings, such as characterization of metallic–metalloid components, non-metallic components, metallurgical reagents, and risk identification, among others, are presented. Implications of potential environmental impacts from the generation of acid rock drainage (ARD) in mine tailings are discussed. Finally, the article concludes that mine tailings are potentially toxic to both communities and the environment, and cannot be considered as inert and innocuous materials; thus, mine tailings require safe, controlled, and responsible management with the application of the most high management standards, use of the best available technologies (BATs), use of best applicable practices (BAPs), and implementation of the best environmental practices (BEPs) to avoid risk and potential socio-environmental impact due to accidents or failure of tailings storage facilities (TSFs).

## 1. Introduction

### 1.1. What Are Mine Tailings? An Initial Scope in an Uncertainty and Complex World

The initial step in mineral processing involves crushing and grinding ore to produce a suitable particle size for further metallurgical processing using physical and chemical separation methods [1]. Physical separation processes take advantage of the different physical properties of mineral particles, such as size, density, and magnetic and surface energy. Commonly used physical separation processes include gravity concentration, magnetic separation, and/or flotation separation, and use jigs, flotation cells, and spirals separator equipment, which may include the use of mining chemicals [2].

Mining chemicals are widely used in mineral processing and the separation of valuable elements. When designing a treatment process, the selection of chemicals is a delicate process that must ensure the most effective separation and concentration results [3].

**Figure 1:**
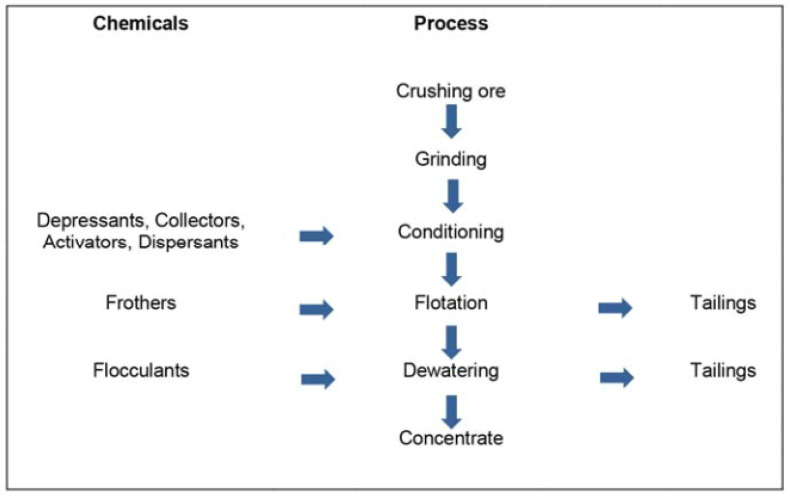
Conceptual diagram of ore processing, concentrate production, and mine tailings generation.

Flotation is the most widely used technique for recovering/extracting minerals from ores. The method is based on the separation of particles from a mixture by altering the conditions of the surfaces of the particles, inducing them to adhere to the air bubbles [4]. Separation by flotation is based on differences in the hydrophobicity of minerals. To separate minerals by flotation, fine air bubbles are introduced into a slurry of finely ground ore, mining chemicals, and water [5]. In the suspension, the chemically modified mineral particles collide with the air bubbles; the minerals that favor contact with the air adhere to the air bubbles and float to the surface [6]. To help in the flotation process, different types of chemical products or reagents can be used: collectors, frothers, pH regulators, modifiers (depressants, activators, and dispersants), and flocculants [7] (Figure 1). The flotation pulp is treated with reagents to change the degree of hydrophobicity of the surface of minerals and create favorable conditions. Reagents are powerful and flexible means that provide the necessary flotation selectivity. Flotation reagents can be of organic or non-organic origin, and can be present in the pulp in molecular or ionic form. The order and form of addition of the reagents depend on the type of sorption and the mechanism of influence on the components of the flotation system. When air bubbles accumulate on the surface of the flotation cell, a foam forms that eventually overflows. This foam corresponds to the concentrate of the metal benefit. In contrast, more hydrophilic minerals remain in the slurry; these materials are called gangue or mine tailings [8].

A first definition that can be mentioned for mine tailings is that they are mining waste materials, there being different types of additional mining waste such as mine waste rock, slag, and leaching pads. Important differences distinguishing mine tailings from the other mentioned mining wastes are: (i) particle size, with mine tailings having a fine granulometry composed of sand, silt, and clay; (ii) the presence of chemical reagents from the metallurgical process; and (iii) management via the use of water for transportation. Mine tailings may or may not be mixed with the process water that remains after the metallurgical treatment of ore, ore concentrate, or extracted materials to obtain marketable components such as metals or minerals [9]. Mine tailings material may contain ground rock, sand, clay, chemicals from the metallurgical process, and residual metals, minerals, and sulfides [10].

**Figure 2:**
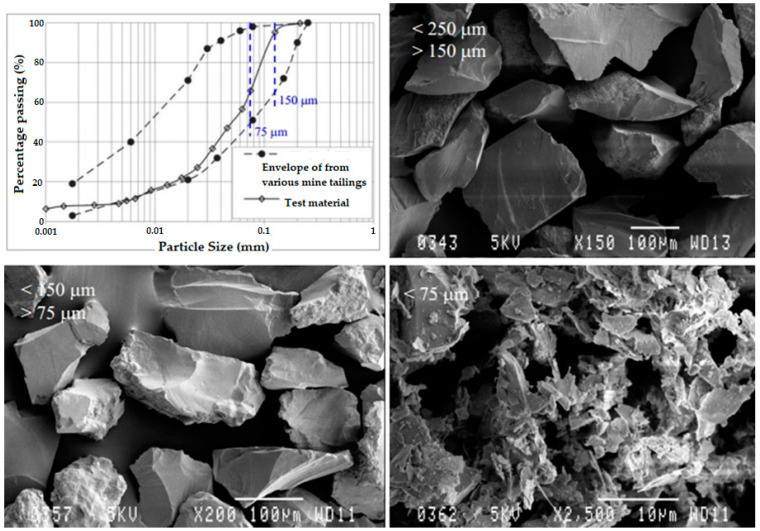
Typical particle size distribution curve and electronic microscope image of mine tailings.

In practice, there are different definitions for mine tailings from different points of view considering the experience of different professional disciplines involved in mine tailings management [2]. Thus, for example, from a metallurgical point of view and considering the process, mining tailings are defined as the remaining low-grade material of valuable beneficiation metal, that is, the gangue obtained through the flotation process [11]. Another discipline that intervenes in the issues of tailings transport from the concentration plant to the tailings deposit is the hydraulic area, where tailings are defined as a solid–liquid mixture, whether a slurry, paste, or solid in bulk, with a concentration of solids determined by weight (Cw) associated with a viscous hydraulic behavior with rheology of the Newtonian or non-Newtonian type [12]. When the mine tailings are deposited in the tailings storage facility, another discipline intervenes, geotechnics, where mine tailings are defined as sand, silt, and clay-type particles with an average size of 75 µm in diameter, with little to no cohesion, and with an angle of internal friction of the order of 35° (see Figure 2) [13]. In this figure, it is possible to appreciate the heterogeneity in the shape of the particles that are part of the mine tailings. Angular shapes and edges are observed, which explains the high abrasiveness of mine tailings present in hydraulic transport by pipes or flumes. This is due to the crushing and grinding processes that are applied to the ore, and is strongly influenced by the stages and types of grinding, whether ball grinding or SAG grinding. Finally, another discipline that is involved in managing mine tailings stored in the tailings storage facility is geochemistry, where tailings are defined as materials with a high diversity of chemical elements due to their mineralogy and prone to changes due to the occurrence of chemical reactions with the environment, such as hydrolysis, oxidation, and leaching, in certain cases leading to the generation of acid rock drainage (ARD) [14].

**Figure 3:**
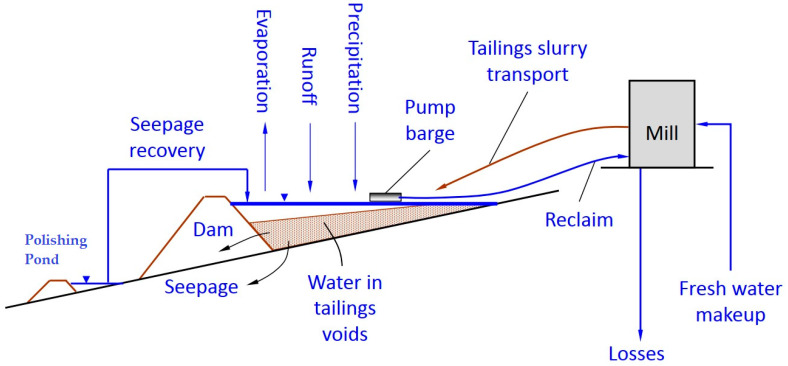
Schematical view of water management in a tailings storage facility.

Mine tailings must be stored in a safe and controlled manner in a tailings storage facility where there is a containment dam and a reservoir area for the storage of these mining wastes [15]. In addition, it is important to highlight that the management of water that has come into contact with the mine tailings, called process water, must be managed efficiently to avoid any type of spillage into the environment [16]. Process water is usually recovered and recycled during the grinding and flotation metallurgical processes, as shown in Figure 3.

**Figure 4:**
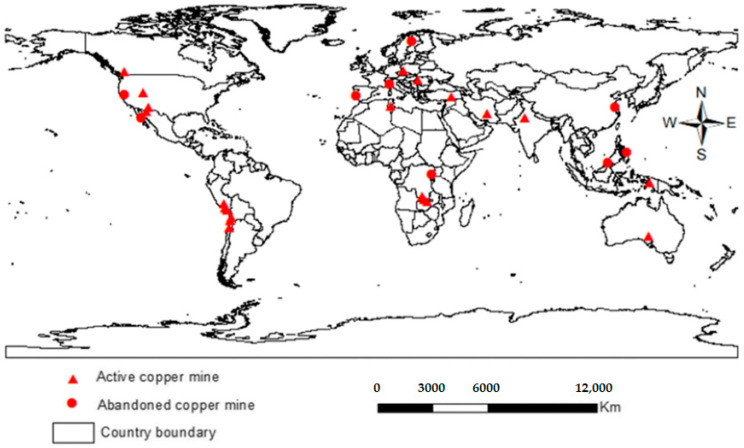
Location of the most representative copper mines in the world [20].

Many of the studies related to the socio-environmental risk of mine tailings in the world state that the impacts on health and the environment are latent, and in many cases bioaccumulation of heavy metals in tissues of organisms and in humans has been demonstrated. In the case of copper mine tailings, investigations have shown that there is a high risk to health [17]. In the countries with the highest copper production, Chile and Peru, research has shown that contamination by mine tailings is significant for the health and environment of the surrounding communities due to a lack of adequate management of tailings and mine closures [18,19]. Similarly, the countries with the largest environmental footprint in copper production are the United States, China, and Canada. A systematic review carried out by Punia (2021) [20] showed the location of all active and abandoned mines with high risk in the world. These risks are associated with environmental factors such as temperature, wind, and precipitation and their effects on the dispersion of contaminants, especially from copper mine tailings (Figure 4).

**Figure 5:**
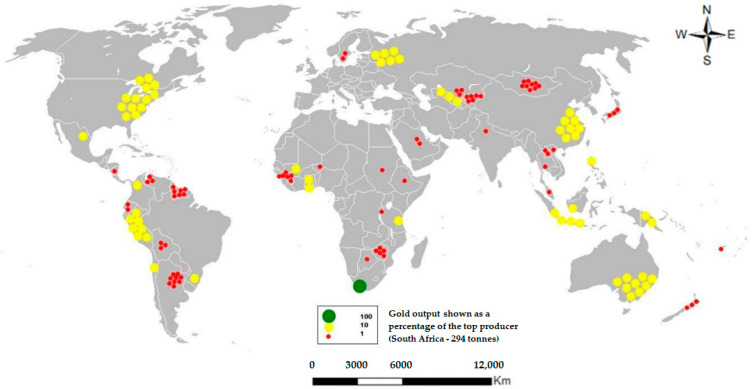
Location of the most representative gold mines in the world.

Gold mine tailings pose a risk to health and the environment as well. One of the historical places with the highest gold production in the world, South Africa, has reported a direct relationship between the presence of heavy metals in soil and water and cases of cancer in children and adults in places surrounding tailings storage facilities [21]. In certain places, especially those involved in informal and artisanal mining, mercury amalgamation is used to separate gold. This heavy metal is highly toxic to life, especially when present in soils, aquatic spaces, and drinking water [22,23]. In the case of cyanide usage in gold leaching processes, it is mainly used in large-scale mining. The inadequate management of this chemical element can generate a series of environmental problems during this process as well as in the generation of mine tailings. Mine tailings from gold mines lead to the accumulation of heavy metals, acid rock drainage, dust emission, and surface runoff, with considerable effects on agricultural spaces, human health, and ecosystems, according to cases reported in China and South Africa [21,24]. The countries that currently have gold mining and mine tailings generation are: South Africa, United States, Mexico, Russia, Peru, China and Australia (Figure 5).

After the removal of valuable minerals carried out during the flotation process, the remaining liquid–solid residues, that is, mine tailings, are often alkaline, having an initial pH between approximately 9.5 and 12.0 [25]. As mine tailings age and the solids react with liquids and air, the liquid pH can become acidic over time, with the potential for ARD [26]. However, this risk must be contextualized considering the massive amounts of minerals that are processed due to lower grades in mining deposits and the enormous amounts of chemical reagents that are ultimately used in the process [27].

Mine tailings can contain potentially hazardous metals found in the original ore as well as organic and inorganic chemical residues from mining reagents used in extraction processes [28,29,30,31]. Therefore, mine tailings are often a high potential source of contamination, both during the operation stage and in perpetuity after the mine closure stage [32,33,34,35].

### 1.2. Methods for Disposing of Mine Tailings

**Figure 6:**
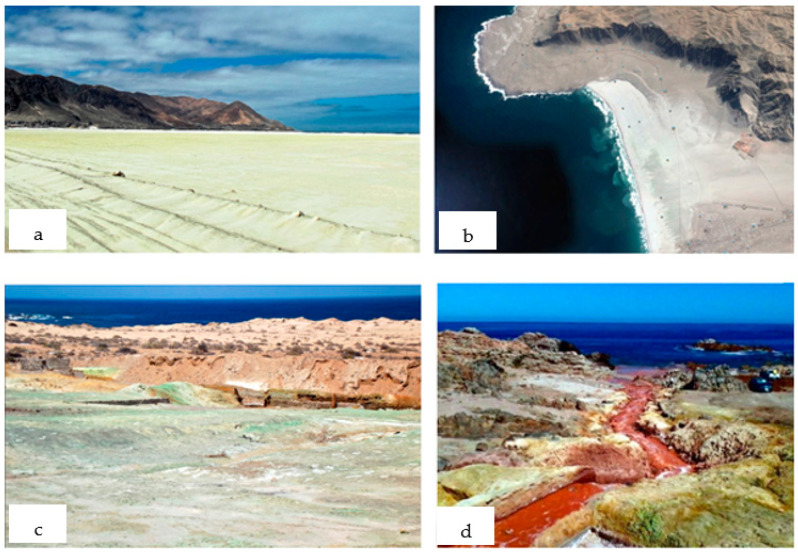
Mine tailings disposal in Chañaral Bay, Chile—Discharge of mine tailings to Salado River and final disposal in the sea. (**a**) Panoramic view of mine tailings beach in Chañaral Bay, (**b**) aerial view of Chañaral Bay with mine tailings, (**c**) mine tailings with oxidation, and (**d**) discharge point of mine tailings and process water to Caleta Palitos Bay.

**Figure 7:**
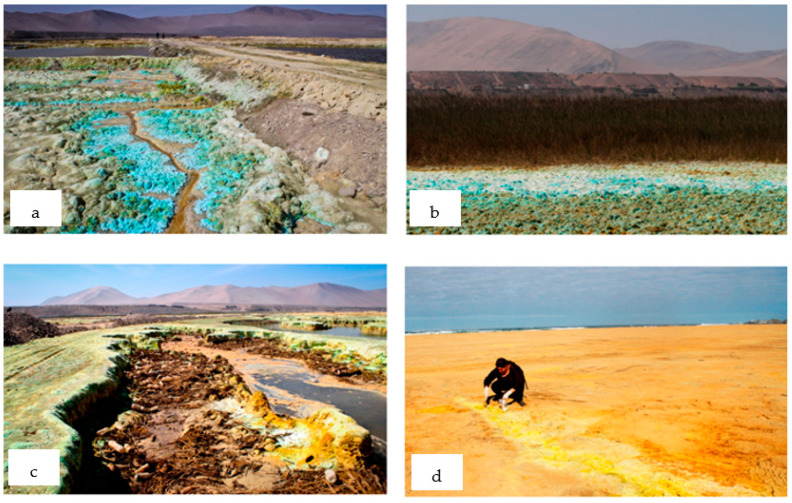
Mine tailings disposal in Ite Bay, Peru—Discharge of mine tailings to Locumba River and final disposal in the Sea. (**a**) Panoramic view of mine tailings beach in Ite Bay, (**b**) presence of copper in mine tailings, (**c**) Ite Bay beach with mine tailings with impacted ecosystems, and (**d**) mine tailings with oxidation.

Mine tailings have historically been deposited in different ways, mainly focusing on the criteria of making transport, deposition, and storage as cost-effective as possible [2]. In the last century, when there were no environmental regulations, mine tailings were dumped into rivers, causing mine tailings migration to the sea [36,37,38,39]. In this way, a part of the mine tailings was deposited in the bed or banks of the rivers, on the beaches along the coast, and at the bottom of the sea [40]. There are two cases in South America where this technique was applied: the cases of Bahía de Chañaral in Chile [10] (Figure 6) and Bahía de Ite in Peru [41] (Figure 7). In both cases, the communities continue to perceive the environmental impacts caused by the use of this technique today. The beaches along the coastline store enormous amounts of mine tailings that are dispersed by the winds and oxidized by the air and seawater, generating acid rock drainage (ARD) in the environment [10,14,41]. This can be seen in the image’s shades of green and yellow-orange colors on the beaches, mainly due to the presence of copper and iron, respectively, which emerge due to the chemical reactions of hydrolysis and leaching that occur in the tailings over time when outdoors.

In the 1990s, a series of environmental regulations were promulgated which prohibited the discharge of mine tailings into rivers and their disposal at sea in both Chile and Peru [2]. This is why today most tailings storage facilities are located on the surface, with dams built to contain mine tailings on geographical surfaces with mountains or hills, mainly in valley-type basins [10,32].

**Figure 8:**
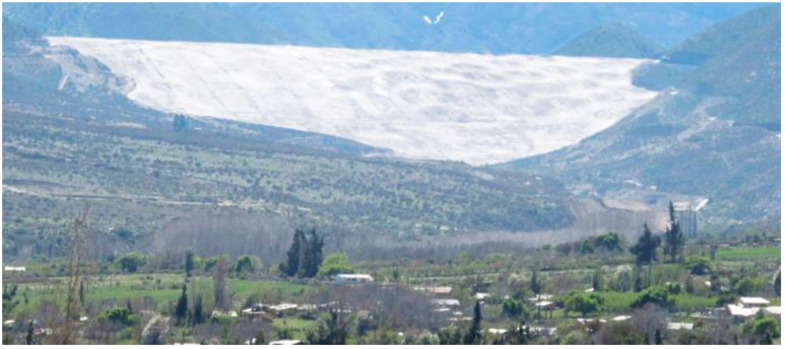
Example of mine tailings disposal in a valley: Cycloned Tailings Sand Dam, Chile, 200 m in height.

Considering mining operations with mine tailings production on the order of 100,000 to 200,000 mtpd, generated 24 h a day and almost 365 days a year, the dimensions of these containment dams can reach heights of over 100 m, in some cases reaching 200 m, and future designs project heights of almost 300 m [16] (Figure 8).

Among the materials used to build tailings storage facility dams are: (i) cycloned tailings sands, (ii) borrowed materials, and (iii) rockfill material from mine waste [2]. Usually, surface tailings storage facilities are located closest to the metallurgical plant in order to save costs in the transportation of mine tailings; in cases where this is not possible, sophisticated transportation systems are built, consisting mainly of pipelines (steel or HDPE) or concrete channels to convey mine tailings to the tailings storage facility [11]. Surface tailings storage facilities can use large amounts of territory, from a few tens of km^2^ to as much as 50 km^2^ [11].

Finally, it should be mentioned that another mine tailings disposal method that has been very popular and attractive in recent decades is storing mine tailings inside underground mines [8]. In this way, it is possible to store mine tailings underground either as hydraulic fill, cemented hydraulic fill, or paste fill. In this way, galleries and stopes can be filled inside the underground mine, allowing the exploration of old pillars of the underground mine, avoiding the storage of mine tailings abroad on the surface with large dams of containment, and considerably reducing the risk of generating acid rock drainage (ARD) [3].

### 1.3. Aim of the Article

There is a need to define mine tailings in a clear, precise, multi-disciplinary, trans-disciplinary, and holistic way, considering geotechnical and hydraulic concepts while integrating environmental and geochemical aspects with implications for the sustainability of mining. This article presents an independent study that answers the questions of what mine tailings are and what socio-environmental risks are linked with mine tailings and their chemical composition. The article considers the practical experience of industrial-scale copper and gold mining projects in Chile and Peru.

Detailed data on the chemical compositions of mine tailings is rarely disclosed to communities and the general public. Occasionally, technical papers may provide selected details on the metal–metalloid components; however, these almost never include complete details on the organic and inorganic chemical components and their concentrations present in the mine tailings.

This article has been structured under the following chapters: (i) introduction; (ii) description of the mining processes linked to mine tailings generated from copper and gold mining on an industrial scale in Chile and Peru; (iii) implications of socio-environmental risks linked to the chemical composition of mine tailings; (iv) presentation of the risks of toxicity in mine tailings due to metals, heavy metals, and cyanide; (v) discussion; and (vi) conclusions. In addition, the implications of potential environmental impacts due to the generation of acid rock drainage (ARD) in mine tailings are discussed.

## 2. Processing of Sulphide Ores and Mine Tailings: Management Experiences from Chile and Peru

### 2.1. Copper Mine Tailings

#### 2.1.1. Metallurgical Process

In copper sulfide mining deposits, whether from surface mining (open pit) or underground mining, the first stage of mineral extraction consists of blasting. After the blasting is done, there are rock fragments that must be loaded into mining trucks with a load capacity of 30 to 300 tons for underground mining and surface mining, respectively [42]. These trucks transport the rock to crushing equipment, where the rock is crushed and reduced in size, where there may be two or three crushing stages. After the rock is crushed in the crushing equipment, it is transported by means of conveyor belts to the concentrator plant. At the concentrator plant, the crushed rock is stacked in a covered stockpile to avoid the emission of particulate matter. The crushed rock material leaves the stockpile into the concentrator plant to start the metallurgical beneficiation process through apron feeders. Then, it is taken to the grinding equipment where water is injected into the crushed rock, forming a mineral pulp. There are usually two grinding stages, namely, grinding in SAG mills and grinding in ball mills. When the grinding stage is finished, the mineral pulp is conducted through hydrocyclones, hydraulic pumps, and pipes to the flotation cells [1,43].

The function of the flotation area is to recover beneficiation metal into a copper concentrate while rejecting coarse, non-sulfide gangue to first stage tailings and rejecting zinc, lead, and iron sulfides to the tailings stream from the second stage [2].

Lime is added to the grinding circuit to ensure that the roughing circuit pH is maintained at its set value of 10. The flotation process typically consists of two copper roughing flotation benches, each consisting of seven forced-air mechanical flotation cells. These produce a coarser low-grade concentrate containing copper, silver, gold, molybdenum, and other non-valuable gangue and sulfide minerals (mine tailings) [44].

The copper cleaning circuit consists of three cleaning stages and one stage of cleaning and removal flotation cells. The first stage of the cleaner typically consists of four forced-air flotation cells, the second typically consists of three forced-air flotation cells, and the cleaner-sweep consists of six cells. The third cleaners are made up of two column flotation cells, each 5 m in diameter by 12 m in height and equipped with washing water [45].

**Figure 9:**
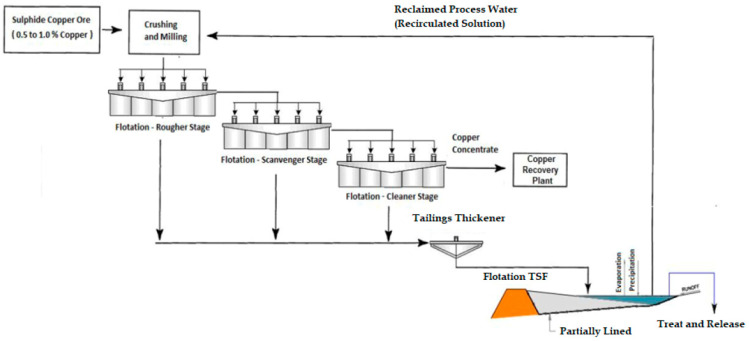
Typical sulphide copper ore flowsheet process of copper mine tailings management.

The flow chart is a standard upstream configuration with sweep. Intermediate concentrate from the first scrubber is reported to the second scrubber feed sump, while tailings are reported to the scrubber-disposal stage. The cleaner–scrubber concentrate is returned to the crusher and the cleaner–scrubber tailings are combined with the coarser tailings and report to the tailings thickener. The second cleaner concentrate informs the third cleaner, and the third cleaner concentrate informs the copper concentrate thickener. Tailings from the third cleaner report to the second cleaner and tailings from the second cleaner report to the first cleaner. The generated mine tailings flow by gravity to the tailings thickener for water recovery and final disposal in the TSF [9] (Figure 9).

After the mine tailings are thickened in the thickening equipment, they are generally transported to the tailings storage facility hydraulically, either in pipe systems or concrete flume systems [11]. In the case of pipelines, mine tailings can be transported by gravity or by using high-pressure pumping equipment. When flume systems are implemented, the tailings flow through the action of gravity [27]. In the case of copper mine tailings obtained by flotation, it is not mandatory to install geomembrane-type geosynthetic systems on the entire surface of the TSF reservoir. Geomembrane liners are usually installed on the slope upstream of the TSF dam in order to avoid seepage through the core of the dam, which risks the occurrence of liquefaction or piping phenomena [16,46].

**Figure 10:**
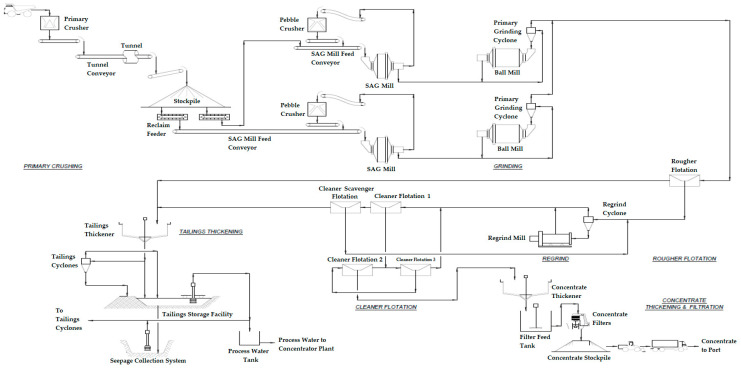
Typical detailed sulphide copper ore flowsheet process of copper mine tailings management.

**Figure 11:**
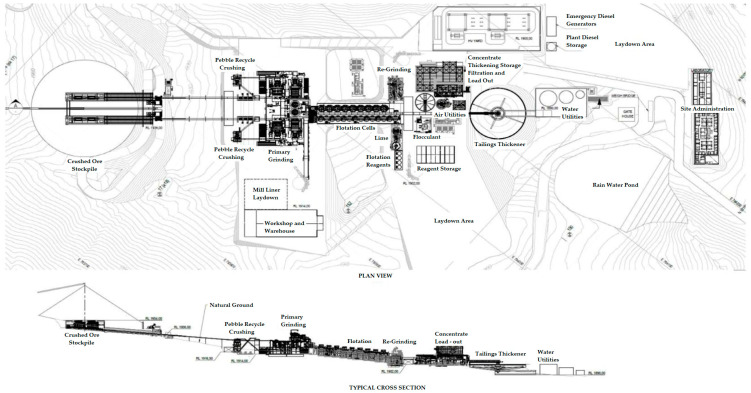
Typical layout and cross-section view of sulphide copper metallurgical plant.

In Figure 10 and Figure 11 it is possible to see a global flow diagram of the metallurgical process of copper sulfide type minerals and a plan view showing a typical cross-section view of the mineral concentrator plant, respectively.

When the mine tailings are deposited in the TSF, the phenomenon of sedimentation and consolidation of the mining tailings occurs, where the solid–liquid separation of the solid part of the mine tailings from the process water occurs, forming a supernatant process water pond [16,27]. In the aforementioned pond, floating barges with pumping equipment are installed in order to recover process water and lead it back to the concentrator plant for reuse in the grinding, flotation, and transport processes of the mine tailings [12].

It should be mentioned that as a result of carrying out this metallurgical process in copper sulfide-type minerals, 3% per ton of processed ore becomes copper concentrate and the remaining 97% corresponds to mine tailings [7].

**Figure 12:**
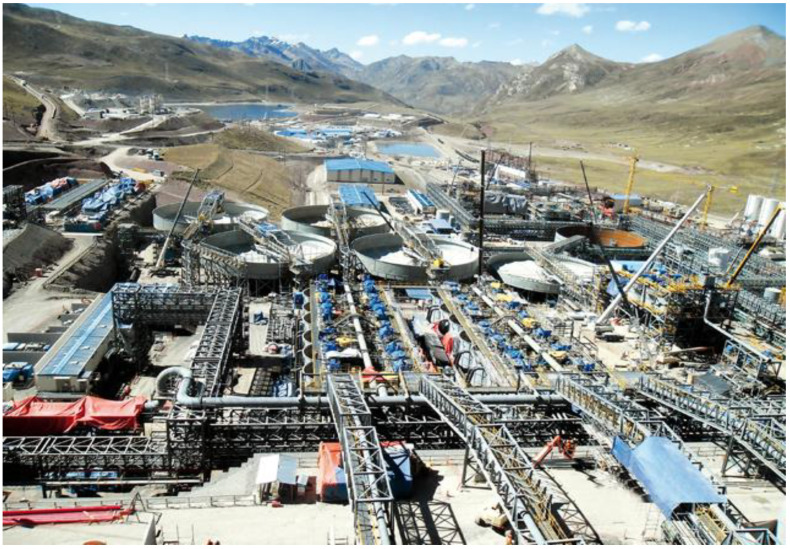
Typical view of sulphide copper concentrator plant showing flotation cells, tailings thickener, and concentrate thickener.

Finally, Figure 12 shows a typical panoramic image of the facilities of a metallurgical plant for the processing of copper sulphide minerals, where the flotation cells and the mine tailings thickeners can be seen.

#### 2.1.2. Typical Metallurgical Reagents Present in Copper Mine Tailings

The main chemical reagents used in the metallurgical flotation process of copper sulphide minerals and present in copper mining tailings are presented below [2,47]. The following types of reagents are disclosed: (i) acids, (ii) alkalis or pH regulators, (iii) modifiers, (iv) collectors, (v) foaming agents, and (vi) flocculants (Table 1).

Cyanide is used in the metallurgical copper recovery process as a flotation reagent called sodium cyanide, with the aim of separating copper from other metals in ores. When used for flotation purposes, as in this case, cyanide concentrations are generally much lower than those used in leaching facilities such as heap leaching or tank leaching [48].

On the other hand, the flocculants most used in the mining industry are synthetic, specifically copolymers of acrylamide groups and sodium acrylate. These groups can have different behaviors depending on the system due to the adsorption mechanisms and function of each group and to the ionic charge of the polymer. The type of flocculant and the dose in which it is supplied must be carefully chosen to ensure that the resulting flocs have adequate density to minimize the energy consumption of transporting the mine tailings to the TSF while maximizing the recovery of water from the supernatant process to be recirculated to the concentrator plant. Over the next few years, an increase in the consumption of flotation reagents and flocculant reagents in the mining industry is expected due to the increase in exploitation projects of mining deposits [11].

#### 2.1.3. Typical Metals and Metalloid Compounds Present in Copper Mine Tailings

The main metallic and metalloid chemical elements present in copper mine tailings are presented below in Table 2 and Table 3, respectively. The chemical symbology and a brief description of each chemical element are presented.

The most dangerous chemical elements for living beings and humans present in copper tailings are Arsenic, Lead, Cadmium, and Uranium [49,50,51,52]. If the intake dose of these chemical elements is high, there is a risk of effects due to toxicity [53]. Later in this article, additional aspects of socio-environmental impacts, effects on human health and toxicity of these elements are exposed.

#### 2.1.4. Typical Non-Metal Compounds Present in Copper Mine Tailings

The main non-metallic chemical elements that can be found in mine tailings are presented below, mainly chlorides, sulfates, nitrates, and ammonium specifically dissolved in the supernatant process water from the tailings storage facility [7,16,27] (Table 4).

These non-metallic elements present in the supernatant process water from tailings storage facilities can migrate towards aquifers and rivers of a hydrographic basin due to the phenomenon of seepage and percolation, producing an alteration of the chemical quality of groundwater and surface areas neighboring the tailings storage facility [39,54,55].

**Figure 13:**
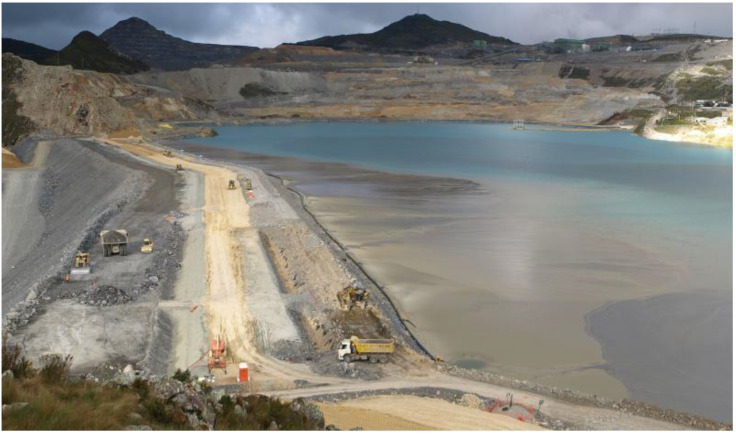
View of copper tailings storage facility located close to concentrator plant, showing conventional use of tailings.

Figure 13 shows a typical view of a copper tailings storage facility using conventional tailings technology located in the Andes, a typical landscape for copper mining projects located in Chile and Peru.

### 2.2. Gold Mine Tailings

#### 2.2.1. Metallurgical Process

In gold sulfide mining, deposits are usually exploited with the underground mining methodology, and to a lesser extent with open pit mining. The first stage in achieving mineral extraction consists of blasting with explosives. After the blasting is finished, the resulting rock fragments are loaded into 30-ton capacity mining trucks or cargo trains. These transport systems carry the rock to the crushing equipment, where the rock is crushed and reduced in size; there may be two or three crushing stages. After the rock is crushed in the crushing equipment, it is transported by means of conveyor belts or belts to the concentrator plant. At the concentrator plant, the crushed rock is stacked in a covered stockpile to avoid the emission of particulate matter. The crushed rock material leaves the stockpile and moves into the concentrator plant to start the metallurgical beneficiation process via the apron feeders, and is then conducted to the grinding equipment, where water is injected into the crushed rock and a mineral pulp is formed. There are usually two grinding stages, namely, grinding in SAG mills and grinding in ball mills. When the grinding stage is finished, the mineral pulp is conducted through hydraulic pumps and pipes to the flotation, leaching with activated carbon and cyanide, and counter-current decantation (CCD) thickening processes [56].

**Figure 14:**
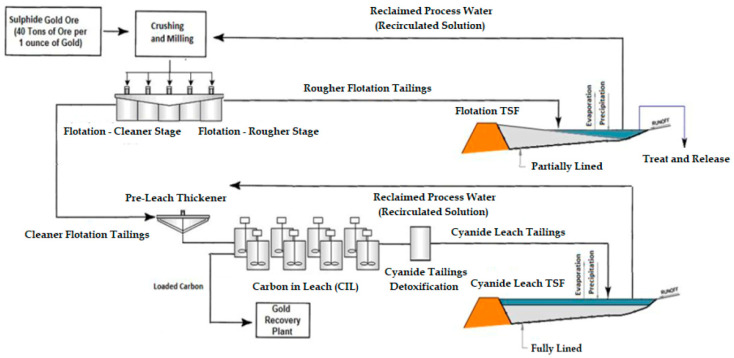
Typical sulphide gold ore flowsheet process of gold mine tailings management.

The flotation process concentrates the gold to approximately ten percent of the original ore mass for cyanide leaching, while the remaining ninety percent is sent to TSF as non-cyanide flotation mine tailings free of most of the minerals from the sulfide present in the ore. For this reason, flotation mine tailings should be stored in separate tailings storage facilities from mine tailings obtained from cyanide leaching [7] (Figure 14).

Because the flotation process is physical, the mine tailings solution is expected to be composed of elements found in the source water used for the process, residual flotation reagents, calcium from lime addition, and any easily soluble salts of the mineral [57]. Mine tailings solids are reduced in sulfide minerals relative to mineral materials, and as such are likely to have limited acid rock drainage (ARD) generating potential on an exact case-by-case basis [58,59].

In the case of mine tailings leached with cyanide, it is possible that they may have high acid (ARD) generation potential. If they are deposited in the form of slurry, this may require the application of techniques to reduce this potential, for example, keeping them completely flooded or underwater using a supernatant process water pond [60,61,62].

**Figure 15:**
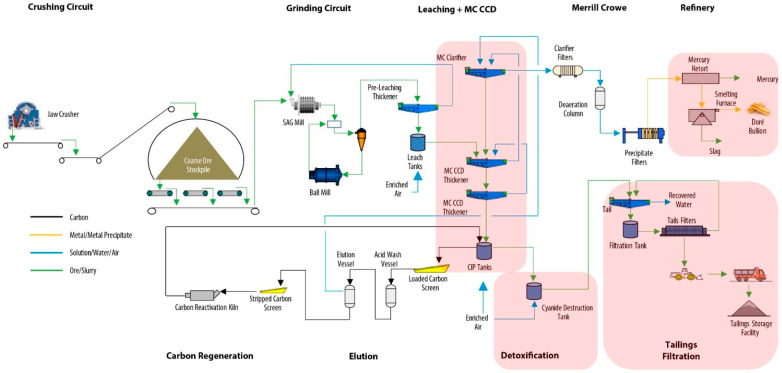
Typical detailed sulphide gold ore flowsheet process of gold mine tailings management.

The concentrate produced by the flotation process is subjected to cyanide leaching to recover the gold. The cyanide process involves dissolving gold (and silver present in soluble form) from ground ore in a dilute cyanide solution (usually NaCN or KCN) in the presence of lime and oxygen. The typical method is to use the counter-current decantation (CCD) thickening and carbon-in-leach (CIL) settling processes. Carbon is added to the ore along with cyanide, air, and lime, and the gold is recovered in the carbon as it is leached by the cyanide [63] (Figure 15).

**Figure 16:**
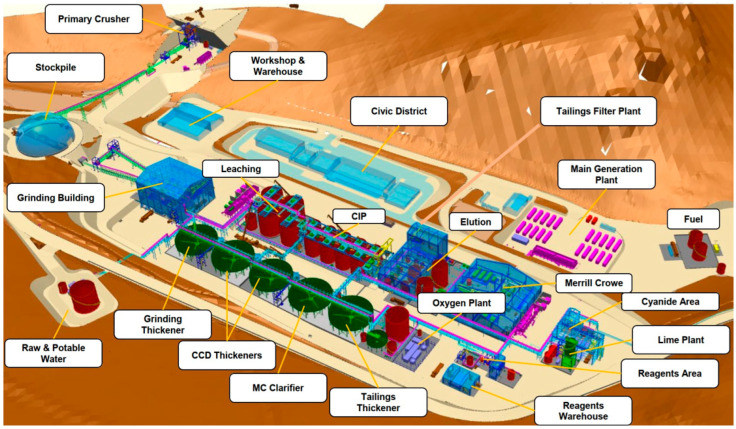
Typical panoramic view of sulphide gold metallurgical plant.

After recovering the gold from the cyanide solution, the leach mine tailings slurry is processed to destroy residual cyanide. The most common destruction method is to use a sulfur dioxide (SO_2_) air cyanide destruction circuit (commonly known as the INCO process) to reduce cyanide concentrations prior to discharge to the TSF [48]. The INCO process is based on the conversion of free cyanides and WAD to cyanate using a mixture of SO_2_ and air in the presence of a soluble copper catalyst at a controlled pH (Figure 10). In the INCO process, different forms of cyanide are removed by different processes. One process involves the conversion of free cyanides and WAD to cyanate. Complex iron cyanides are reduced to the ferrous state and precipitate as insoluble copper–iron–cyanide complexes. Residual metals released from WAD cyanide complexes precipitate as their hydroxides [64] (Figure 16).

In addition, it should be mentioned that as a result of carrying out all this metallurgical process in gold sulfide-type minerals, only one to two ounces of gold are extracted per ton of ore, generating significant amounts of mine tailings [65].

Free and weakly complexed metal cyanides (i.e., WAD cyanides) are oxidized to cyanate by sulfur dioxide and air in the presence of a soluble copper catalyst.

CN^−^ + SO_2_ + O_2_ + H_2_O Cu Catalyst = OCN^−^ + SO_4_^−2^ + 2H^+^ (1)

M(CN)_4_^−2^ + 4SO_2_ + 4O_2_ + 4H_2_O Cu Catalyst = 4OCN^−^ + 8H^+^ + 4SO_4_^−2^ + M^+2^ (2)

The SO_2_ required in the reaction can be supplied as liquid sulfur dioxide, sodium sulfite (Na_2_SO_3_), or sodium metabisulfite (Na_2_S_2_O_5_). The reaction is normally carried out at a pH of about 8.0 to 9.0; due to acid formation in the reactions, lime is normally required for pH control. Decreases in process performance may occur if the pH fluctuates outside of this optimum range. The optimum pH must be developed experimentally, as the maximum removals of cyanide and metals occur at different pH values [48,63,66].

After the mine tailings are generated, if they contain high levels of cyanide they must be subjected to the cyanide destruction process. In addition to carrying out this process, many mining companies seek to avoid running the risk of depositing gold mine tailings as slurry in the TSF, because in this case seepages with cyanide contents can be produced, altering the chemical quality of aquifers in hydrographic basins [67]. For this reason, in addition to applying the cyanide destruction process to reduce the amount of cyanide present in the mine tailings, tailings filtering technology is applied to recover the maximum amount of process water and part of the remaining cyanide. In this way, the mine tailings to be deposited in the TSF consist of a dry bulk material with low moisture content and reduced amounts of cyanide [13].

As explained above, gold ore treatment metallurgical plants may prefer to deposit the filtered tailings with band, presses, or ceramic discs filters in dry stacking tailings storage facilities to minimize the percolation of cyanide solutions into the groundwater, especially when considering geological faults and hydrographic basins [68].

In other gold ore treatment metallurgical plants, counter-current decantation (CCD) thickener circuits may be considered to recover the solutions; however, the restrictions imposed on conventional wet tailings storage facilities or those with a lot of supernatant process water favor the use of tailings filtering equipment, where the cake obtained from the filtration contains less than 20 ppm of CN trapped inside [13]. After the previous thickening stages, the filtration stage of the mine tailings through band-type vacuum filters washes most of the solutions containing gold and cyanide in the cake; for this reason, more than 50% of the area used for filtration is dedicated to washing the cake to recover solutions with CN.

**Figure 17:**
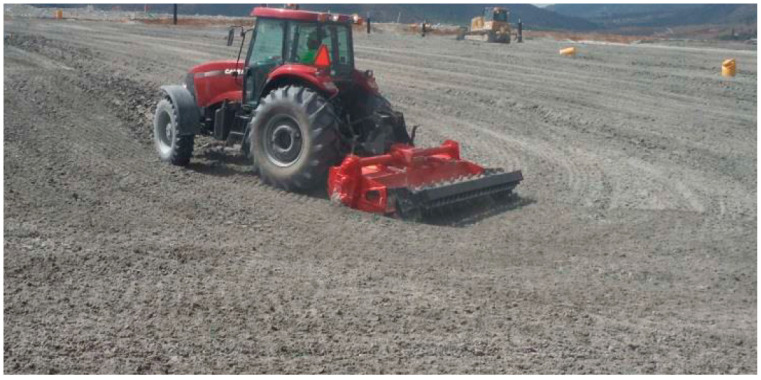
Filtered tailings moved by a plow with nine 24-inch discs to aerate the cake.

After the filtered gold mine tailings are transported to the TSF, either by truck or conveyor belt, they must be deposited in the tailings storage facility in layers of 20 cm compacted with the support of bulldozer-type mechanical equipment and a compacting roller. For safety reasons, a cyanide degradation procedure must be applied to the filtered tailings [42,69]. After the 20 cm layers of filtered tailings have been distributed in each sector of the tailings storage facility, it is possible to wait for the loss of moisture from the cake (approximately 5–10%), which occurs in 14 h without exposure to sun or 4 h with sun exposure. When the optimum humidity has been reached, the mine tailings are moved with a plow with 9 24-inch discs to aerate the cake and facilitate the penetration of water (Figure 17). Airing and wetting of the filtered tailings helps to promote the destruction of cyanide. The water generates hydrolysis of sodium cyanide to hydrocyanic acid, which by the effect of heat volatilizes and oxidizes in the presence of air and heat [70]. The procedure to remove the filtered tailings must be carried out in the early hours of the morning, from 9:00 a.m. to 12:00 p.m.; then at 04:00 p.m. the irrigation of the deposited material is performed sequentially to the removal of the material to promote aeration and change the face exposed to the sun. This process is repeated until reaching 96 h of exposure (or more, if required by weather conditions). When the cyanide degradation period has concluded, the filtered tailings are compacted with the support of a compacting roller. Compaction is produced by the continuous passage of vehicles and heavy equipment on the surface of the filtered tailings, producing a good level of compaction of the surfaces that is available to receive a new layer of mine tailings after four days (or more, if climatic conditions require it). A new layer of filtered tailings is then deposited, which continues until completion of 100 cm of height over the entire area of the filtered tailings storage facility [13].

**Figure 18:**
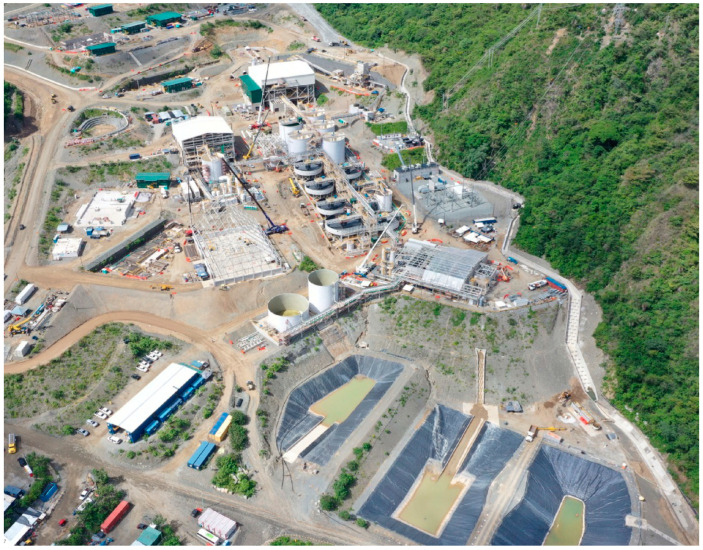
View of sulphide gold concentrator plant, showing gravity concentration, cyanide leaching, counter-current decantation (CCD) thickening, and Merrill Crowe processes.

The following Figure 18 shows a typical image of a metallurgical plant for the processing of gold sulfide minerals where the used chemical reagents and the generated gold mine tailings must be properly managed.

Finally, another alternative to store gold mine tailings with traces of cyanide that has been implemented in mining projects is to dewater the tailings with thickener-supported paste tailings technologies, then use the dewatered tailings as cemented hydraulic fill or backfill in paste form to fill the galleries and stopes of underground mines. This approach avoids depositing gold mine tailings with remnants of cyanide on the surface, either in basins or valleys, eliminating the risk of seepage into underground water flows close to local communities and acid rock drainage (ARD) generation [8].

#### 2.2.2. Typical Metallurgical Reagents Present in Gold Mine Tailings

The chemical reagents used in the metallurgical process of gold beneficiation are similar to those presented for the case of copper; therefore, Table 1 is valid for this case as well.

In gold mining, a dilute cyanide solution is poured over the crushed and ground rock. Cyanide binds with gold and silver (and with other metallic and metal-like elements) to form soluble compounds. Gold and silver are then preferentially mined, leaving the other metals and contaminants behind in the mine tailings. Solutions for the mining process are kept alkaline (typically above a pH of about 9.5) to prevent the formation of deadly hydrocyanic acid gas [71]. A typical dosage of sodium cyanide applied in gold mining considering 2 cases are: (i) flotation and carbon in pulp (CIP) processes is in the range of 150–500 g/ton of ore processed, and carbon in leach (CIL) processes is in the range of 0.25–0.5 g/L of solution [48].

The metallurgical process plant for the treatment of gold ore, specifically in terms of cyanide treatment, must be designed taking into account the requirements of the International Cyanide Management Code, which contemplates the highest world standard in the management of this substance [63].

#### 2.2.3. Typical Metals and Metalloid Compounds Present in Gold Mine Tailings

The main metallic chemical elements present in gold mine tailings are presented below [67] in Table 5, including their chemical symbology is presented. Depending on the mineralogy of the mining deposit, certain gold mine tailings may contain lower or higher concentrations of mercury.

#### 2.2.4. Typical Non-Metal Compounds Present in Gold Mine Tailings

As in the case of copper mine tailings, it is expected that in gold mine tailings there will be concentrations of total dissolved solids of non-metallic compounds corresponding to (i) chlorides, (ii) sulfates, (iii) nitrates, and (iv) ammonia [2,16,27].

The following paragraph provides an estimate of the likely concentration parameters in the mine tailings produced by cyanide leaching of the concentrate followed by destruction of residual cyanide. The concentration ranges for the various parameters are based on data from practical experience of gold metallurgical processing in industrial-scale gold beneficiation mining projects. The process water from gold mine tailings presents high concentrations of total dissolved solids consisting of sulfate (4000 mg/L to 6000 mg/L) produced by the addition of cyanide in the leaching process and destruction of cyanide in the tailings [67,70]. On the other hand, the sodium concentrations are on the order of 2000 mg/L as a result of the addition of cyanide in the leaching process and destruction of cyanide in the mine tailings. In addition, added calcium is found for pH adjustment during leaching and during cyanide destruction as well as nitrogen compounds (total 100 mg/L nitrate, ammonia, CNO, nitrite) from cyanide destruction [48]. It is possible that metal concentrations could be problematic depending on the trace metal content of the ore.

To remove sulfate and bring elevated concentrations down to a target concentration of 500 mg/L or less, the use of reverse osmosis (RO) is required [61]. High-pressure membrane processes such as reverse osmosis (RO) are typically applied for the removal of dissolved constituents, including organic and inorganic compounds. RO is a process in which the mass transfer of ions across membranes is controlled by diffusion. Consequently, these processes can eliminate salts, hardness, synthetic organic compounds, disinfection by-product precursors, etc. However, dissolved gases such as hydrogen sulfide (H_2_S) and carbon dioxide, monovalent ions such as chlorine (Cl) and sodium (Na), and certain pesticides are able to pass through RO membranes [64,72].

On the other hand, mine tailings remaining after cyanide leaching and destruction may be classified as PAG (Potentially Acid Generating), and need to be properly managed at the tailings storage facility to avoid generation of Acid Rock Drainage (ARD) [59,60].

**Figure 19:**
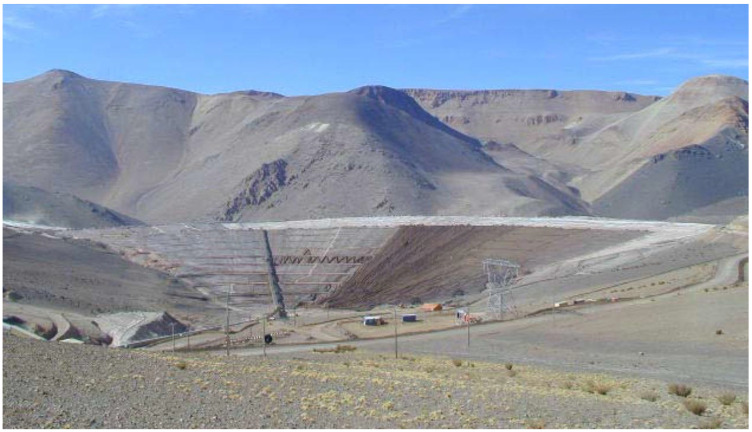
Typical view of gold tailings storage facility showing filtered tailings technology with dry stacking at a mine in the Andes mountains.

Figure 19 shows a typical view of a gold mine tailings storage facility with filtered tailings technology located in the Andes, a typical landscape for mining projects located in Chile and Peru.

## 3. Socio-Environmental Impacts and Risks Generated by Mine Tailings

The following chapter presents an identification and description of the main impacts and socio-environmental risks generated by mine tailings. The concepts and ideas presented in this chapter are the product of practical experience acquired over the years in the mining and metallurgical industry in both Chile and Peru.

### 3.1. Conceptual Model of Socio-Environmental Impacts and Risks Produced by Mine Tailings

To understand the socio-environmental impacts and risks produced by mine tailings in the environment, it is necessary to define two conceptual models: (i) a conceptual model of socio-environmental impacts and (ii) a conceptual model of socio-environmental risks [7].

**Figure 20:**
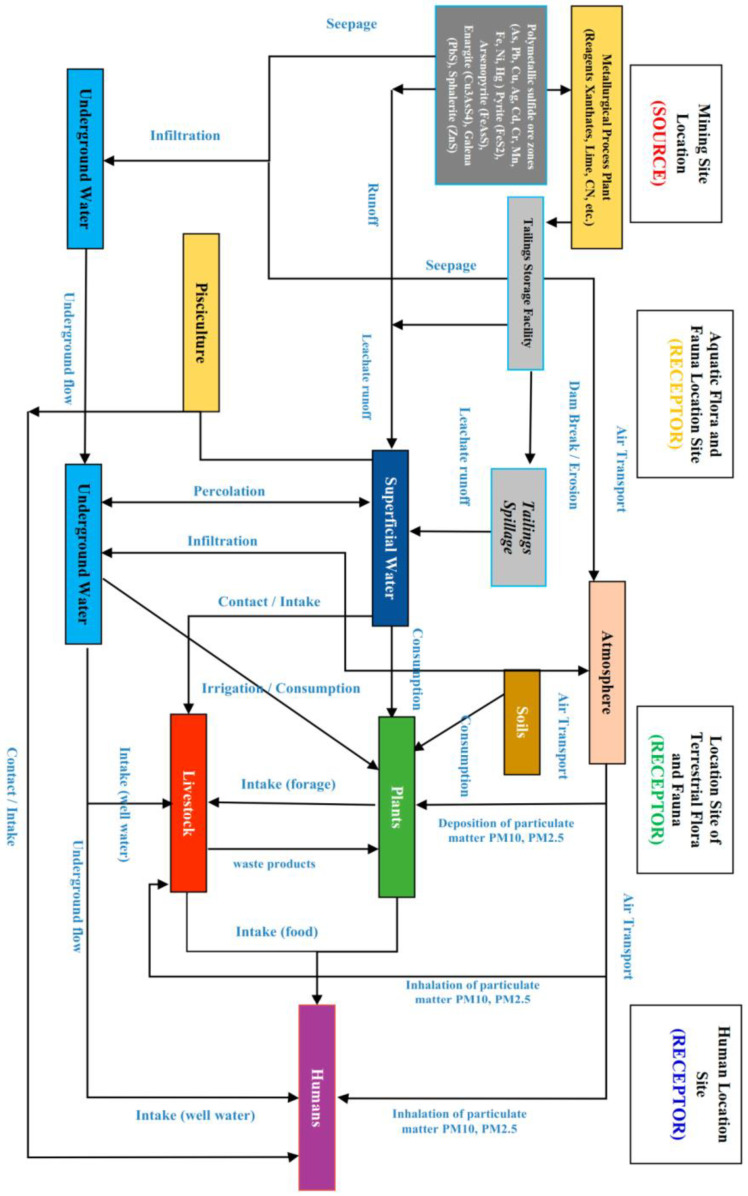
Conceptual Model of Socio-Environmental Impacts from Mine Tailings Spill [7].

Figure 20 illustrates the conceptual model of socio-environmental impacts; it is possible to appreciate how the source of potential contamination affects the environment through different mechanisms of dissemination of contaminants in different media, including a series of receptor organisms (humans, flora, and fauna).

**Figure 21:**
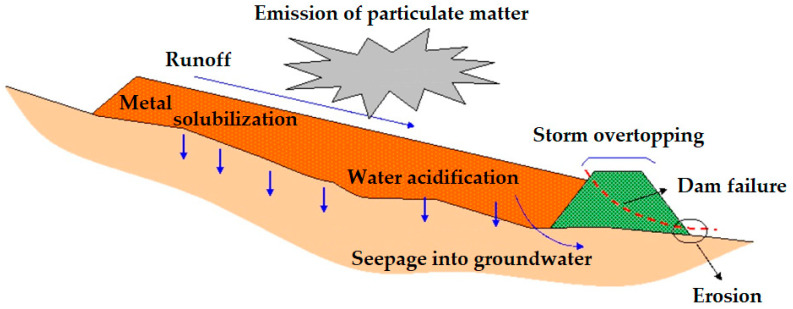
Conceptual Model of Potential Risks from Mine Tailings Storage Facility.

On the other hand, Figure 21 shows the conceptual model of socio-environmental risks potentially be generated due to irresponsible and unsafe management of mine tailings.

In the analysis of the socio-environmental risks of mine tailings considering their chemical composition, it is necessary to define and clarify a conceptual model for adequate management of the affected spaces and their surroundings. Figure 20 presents a model focused on incident factors in the socio-environmental impacts generated by mine tailings. These factors correspond to: (i) sources of contamination; (ii) identification of relevant polluting material, its spatial and temporal distribution in the environment (air, soil, and water) along with its chemical properties (speciation); (iii) its mobilization mechanisms; (iv) possible routes of exposure; and (v) the potential receptors of the contamination. Based on these data from the affected area and adjacent places, it is necessary to identify the pollutants with the highest probability of presence in potentially affected spaces while focusing on those pollutants with the greatest risk to human health and ecosystems. The spatial and temporal distribution of pollutants depends largely on the environmental characteristics (temperature, moisture, precipitation, wind) and the morphological and edaphological characteristics of the place. When considering the conceptual model (Figure 20), the sediments present in water, soil substrates, and particulate matter in the wind are the means of transport that act as possible sources of contamination and reservoirs of pollutants. Under adequate physical and chemical conditions, they can affect biota and transfer polluting elements to the food chain through bioaccumulation [7].

Tailings storage facilities in general are subject to risks for various reasons, which are altered in magnitude by the danger of their components and by the content of heavy metals, chemical reagents, and their mineralogical characteristics [73,74]. In an illustrative manner, these risks are exposed in a general way above, considering: (i) emission of particle matter, (ii) runoff, (iii) metal solubilization, (iv) water acidification, (v) seepage into groundwater, (vi) storm overtopping, (vii) erosion and (viii) dam failure. The applicability and measurement of each of these risks are defined through a detailed study considering the collection and analysis of samples of surface water, groundwater, vegetation (if any), and air quality.

#### 3.1.1. Impacts and Risks Linked with the Water—Acid Rock Drainage with Leach Contaminants

In a mine tailings storage facility, there are potential risks related to superficial water and hydrogeological water [75,76,77]. This type of risk can give rise to different negative effects on the environment, such as dragging of waste and residues produced by rain, acidification of water due to the effects of tailings, metal solubilization (dissolution of heavy metals in water), sediment entrainment, groundwater pollution, dam overflow in storms, and erosion of the dam base by flow [78,79,80]. These all impact the environment differently.

In the aquatic environment, heavy metals can be very toxic to aquatic life and the human population due to their persistence, toxicity, non-degradability, bioaccumulation, and biomagnification in the food chain [81,82]. Heavy metals can enter the human body through the oral, inhalation, and dermal contact routes [83]. Exposure to high doses of heavy metals can induce complications in the human system, such as nausea, vomiting, diarrhea, kidney failure, neurological changes, Alzheimer’s disease, cardiovascular disease, and cancer [84]. For example, in Peru, studies carried out in the Mantaro River (the main river in the basin that runs through five regions with a strong mining influence) have revealed high concentrations of heavy metals and metalloids that exceed national environmental quality standards [85]. The metals that register relatively high mean concentrations are Pb (9.73 μg/L) and As (14.33 μg/L), both of which exceed the water quality standards of Peru and the threshold values established by the WHO and EPA (10 μg/L) [86].

Another aspect related to the potential impacts and risks linked to water generated by mine tailings is the formation of acid water occurring due to the chemical oxidation of sulfides, accelerated in many cases by bacterial action [87]. The main elements involved are the reactive sulfides present in mine tailings, oxygen, water (vapor or liquid), and a catalyst for bacteria [88].

The behavior of the mine tailings with respect to its chemical stability should be considered in two scenarios: (i) the operation stage and (ii) the post-closure stage of the tailings storage facility [89,90,91,92]. In the operation stage, fresh slurry is continuously deposited in the tailings storage facility; due to the process by which it is produced, this presents alkaline pH characteristics in the range of 9.0 to 11.0, which allows the acid solutions that may be produced in the tailings storage facility to be neutralized [2,12]. It should be noted that tailings slurry has a high water content (30–50% water), and when it is deposited it creates sediments, generating separation of its solid and liquid phases. Thus, the clear waters that cover the mine tailings have a double mitigation effect: on the one hand, the alkaline characteristic of the clear waters allow the neutralization of possible acid solutions that occur in the TSF, and on the other hand, they limit the oxidation of minerals with sulfur content, as a barrier is produced between the deposited mineral and oxygen. Oxygen has low solubility in water, and is an element required by iron-oxidizing microorganisms that allow the oxidation of minerals with sulfur content, such as pyrite, among others [14,87].

On the other hand, during the closure stage of a tailings storage facility, when mine tailings with pyrite are exposed to rain and oxygen for years or longer, acid rock drainage (ARD) is likely to be generated if the mine tailings have a high pyrite content. In light of the above, it is necessary to consider medium- and long-term control measures to mitigate the generation of acid rock drainage (ARD) [60,92].

Most of the oxidation processes that occur in natural environments involve a simultaneous modification of the acid–base state of the system, generally increasing its acidity, as for example occurs in mine tailings over time [93,94]. Pyrite (FeS_2_: iron disulfide) is commonly associated with coal deposits and metal deposits. Oxidation occurs spontaneously in nature and in slurry containing heavy metals in mine tailings, causing environmental problems of the acid rock drainage (ARD) type [26]. This process occurs when large amounts of strongly reduced sediments in relatively small areas that are rich in sulfides from mining activities, sediment recovery processes with sulfides, or other causes are exposed to the weather [10].

In nature, sulphides remain underground in the absence of oxygen, and only a small part of these deposits emerges to the surface. Of all the contaminants of water courses, acid mine drainage is perhaps one of the most serious due to its nature, extent, and difficulty of resolution [26]. The rivers and aquifers affected by this type of contamination are characterized by their acidity, the high content of sulfates and heavy metals in their waters, and the metallic content of their sediments [1].

**Figure 22:**
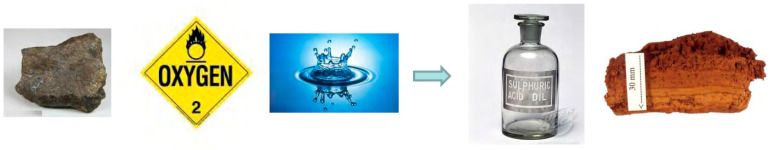
Conceptual image of chemical reaction defining generation of acid rock drainage.

Acid rock drainage (ARD) is water with a high level of acidity and high load of metals in solution. Such acid rock drainage is the result of the reaction of water, both surface and underground, with sulphide minerals [14]. Equations (3) and (4) and Figure 22 provide an example of the chemical reactions that define the generation of acid rock drainage (ARD).

Pyrite + Oxygen + Water = Sulphuric acid + Iron hydroxide(3)

4Fe_2_ + 15O_2_ + 14H_2_O = 8H_2_SO_4_ + 4Fe(OH)_3_ (4)

The oxidation of metal sulfides present in mine tailings is a complex process which includes several types of reactions (oxidation–reduction, hydrolysis, formation of ionic complexes, precipitation solution, etc.) that produce oxidized forms of iron, sulfate anions, and a strong acidity (estimated at two moles of acid per mole of pyrite) [14]. Pyrite exposed to the atmosphere during the excavation and mining processes reacts with oxygen and water to form sulfates that result in acid rock drainage (ARD). This acidity is the product of the action of bacteria of the Acidithionacillus spp type, which generate their energy by oxidizing ferrous ion (Fe^+2^) to ferric ion (Fe^+3^) using oxygen [26]. The ferric ion in turn reacts with the pyrite to generate ferrous ions and sulfates. The ferrous iron is then available for oxidation by bacteria; this cycle continues until the pyrite content is exhausted [88].

Investigations have shown that bacteria of the genus Acidithiobacillus (Acidithiobacillus ferroxidans, Acidithiobacillus caldus) are catalysts for the reactions that occur, and that there are other bacteria involved in this process as well (Acidimicrobium ferrooxidans, Sulfobacillus acidophilus, Leptospirillum ferriphilum, Leptospirillum ferroxidans, Sulfolobus metallicus, Sulfolobus Acidocaldarius, and Thiobacillus thiooxidans) [14,26,41]. The rate of bacterial oxidation depends on factors such as temperature, pH, availability of O_2_ and CO_2_ for cell reproduction, amount of nutrients (N, P), and the surface area of sulphide minerals.

These reactions generate acidity and release large amounts of sulphates, iron, and other metals that sulphides contain (As, Cd, Co, Cu, Pb, Zn, etc.), producing a toxic leachate [26]. This leachate reacts with the surrounding rocks, producing hydrolysis of other minerals and causing elements such as Al, Ca, Mg, Mn, Na, Si, and others to dissolve [14].

**Figure 23:**
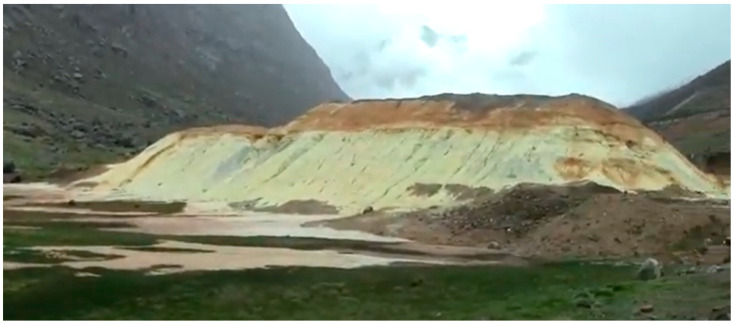
An abandoned tailings storage facility in the Andes mountains showing oxidation of mine tailings.

In Figure 23 it is possible to see an abandoned tailings storage facility without any cover measure, located in a valley of the Andes mountain range; it is exposed to the air and rain in the area, which generates the oxidation of mine tailings visualized through the yellow and brown color of the material. This produces acid rock drainage (ARD) that impacts surface water, groundwater, and local soils.

#### 3.1.2. Impacts and Risks Linked with the Soil—Instability and Accumulation of Metals and Metalloids

Among the potential impacts and risks linked to the soil are those related to the danger of instability due to seismic events [76,95]. This risk is assessed through the physical stability of the slopes of the containment dams and the risk of liquefaction of the tailings storage facility [96,97].

Another relevant point related to soils involves alteration of the chemical quality of soils due to mine tailings [98,99,100]. It is possible for the contents of heavy metals and metalloids to increase, modifying the chemical quality of the substrate (organic matter content) and limiting its use by living beings such as local plants, flora, and fauna. This undoubtedly affects the normal functioning of the ecosystems that are found in the vicinity of tailings storage facilities by altering the ability of plants to carry out photosynthesis, with certain plants being more or less tolerant of high heavy metal contents [101]. In Peru, one of the main economic activities is mining, which has great repercussions in the form of contamination of soils in the high Andes. Studies carried out in mining-influenced areas of the central Andes have revealed Pb concentrations in agricultural soils that vary from 43.70 to 67.30 (mg/kg). These concentrations are below the environmental quality standards of soils in Peru (70 mg/kg) [102]. In the northern Andes of Peru, studies of heavy metals in soils of the Moche River basin have shown variability in their concentrations within the range of what is permitted except in the case of Cu, for which values widely exceed the permitted values [103].

Another important aspect has to do with the use of the land; more than a risk, this is a concrete fact, as the site where the mine tailings are deposited will not return to its initial state in terms of environmental functionality and ecosystem services [104,105]. Thus, the objective is to minimize the impact that land use causes on the environment by having the fewest number of sites that store mine tailings.

Table 6 presents the maximum permissible limits (LMP) and environmental quality standards (ECA) considered for cases of mine tailings spills, whether copper or gold, in the cases of Chile and Peru [7]. In addition, international maximum permissible limits are presented based on the standards of Canada and the FAO, WHO, and EPA.

Finally, it should be mentioned that in the case of Chile there is no standard for regulating the chemical quality of soils, making it necessary to resort to international standards in order to analyze and study the effects of contamination from mine tailings spills. In the case of Peru, regulations consider both water and soil, which makes it possible to assess whether or not there contamination from a mine tailings spill exists [7].

#### 3.1.3. Impacts and Risks Linked with Air—Dust and Emission of Particle Matter

One of the impacts and risks associated with the air is generation of dust. This risk occurs because the particle size of mine tailings makes them prone to wind erosion, causing impacts on the health of people in surrounding communities and direct physical damage to the environment [106]. Particulate material is transported through the air for distances that can reach kilometers in certain cases, transporting heavy metals that can enter the respiratory tract of living beings and bioaccumulate in the lungs due to the small granulometry (on the order of particle diameters of 5.0, 2.5 and 1.0 μm), causing both acute and chronic damage to health [107].

Another aspect little reported in the literature has to do with the bad smells emanating from mine tailings, specifically during the operation stage. Due to the amount of chemical reagents present in mine tailings, strong odors of organic solvents can be perceived by the operators of the mining operation and in neighboring communities. This causes discomfort in people, in some cases producing general and non-specific depression of the central nervous system that translates into headache, nausea, and vomiting, dizziness, instability, vertigo, weakness, loss of consciousness, and even death due to respiratory depression [108,109]. This issue must be considered by mining companies to avoid harm to the health of their own workers and to that of people from neighboring communities [110].

## 4. Mine Tailings Toxicity Caused by Heavy Metals and Cyanide

### 4.1. Toxicity Issues Related to Heavy Metals

Changes in the quality of soil, water, and the atmosphere caused by the presence of heavy metals due to human activities cause changes in the structure and physiology of living organisms, and represent an exposure risk for people living and working in mining areas [111,112]. Mercury, lead, arsenic, and cadmium are non-essential metals for the human organism, and are lethal to humans [113]. Their release into the environment represents a potential risk to human health, as they can enter food chains, persist in the environment, bioaccumulate, and become biomagnified [114]. Heavy metals can enter through skin contact, inhalation, and ingestion. Heavy metals in the liver can be biotransformed, form additional polar compounds, and transform into more active compounds (leading to mutagenesis and carcinogenesis) or less active compounds [115]. Exposure to high concentrations of heavy metals in air, soil, water, or food can lead to autoimmune diseases, allergies, reproductive diseases (interference with the function of the corpus luteum of the ovary, which prepares the mucous membrane of the uterus for implantation of a fertilized ovum), cardiovascular disorders, lung cancer mortality, and overall mortality [116,117,118].

Heavy metals accumulate in adipose tissue, bone, hair, liver, kidneys, and brain, which influences biochemical and hormonal processes such as metabolism, cell proliferation, and fertility, among others [113]. Accumulation of Pb, Se, Hg, As, and Cr in high concentrations can cause toxic effects in the body and lead to death. Pb and Cd are nephrotoxic elements, especially in the renal cortex. Pb exposure in children has an irreversible effect on neurological functions, while chronic exposure in adults causes high blood pressure, damage to the cardiovascular system, neurotoxicity, and cancer development [113]. Adverse effects of As ingestion include nausea, vomiting, abdominal pain, profuse bloody diarrhea, problems related to the kidneys, liver, and skin, and prostate cancer [51]. Cadmium, even more than other heavy metals, is toxic at very low concentrations to plant, human, and animal health. In humans, it causes serious diseases if ingested through the food chain, such as high blood pressure and carcinogenic diseases [93]. Chronic exposure to Cd can cause damage to the kidneys, liver, and skeletal and cardiovascular systems, as well as loss of vision and hearing and development of malignant tumors in the lungs, breasts, prostate, pancreas, urinary bladder, and nasopharynx. Heavy metals disrupt Zn metabolism, resulting in Zn deficiency, which can impair the function of the pituitary, thyroid, and adrenal glands, ovaries, and testicles, thereby reducing fertility [107].

The removal of heavy metals from the soil can be carried out by physical, chemical, and physicochemical remediation technologies to increase the soil pH. Several of these techniques include the planting of plants that exist in symbiosis with microorganisms as phytoremediators coupled with technology that promotes the degradation, containment, or extraction of xenobiotic compounds from the water and soil by various groups of plant species [119]. Likewise, the use of forest plants, mainly native plants that show a high capacity to absorb, accumulate, and adapt to large amounts of heavy metals in their structure, can help to return the soil to a lower state of toxicity [120]. Grasslands used in the revegetation process can be assisted by amendment; this is the most used type of contingency, as it is quick and cheap to implement and considerably able to restore the original ecological and functional properties of the contaminated space [121,122].

Studies have revealed that fauna, especially mammals, are considerably affected, experiencing important alterations due to greater accumulation of heavy metals in organs such as the liver and kidney [123,124]. Likewise, in cases of prolonged exposures of wild animals, heavy metals bioaccumulate until they reach the skin and fur [125].

**Figure 24:**
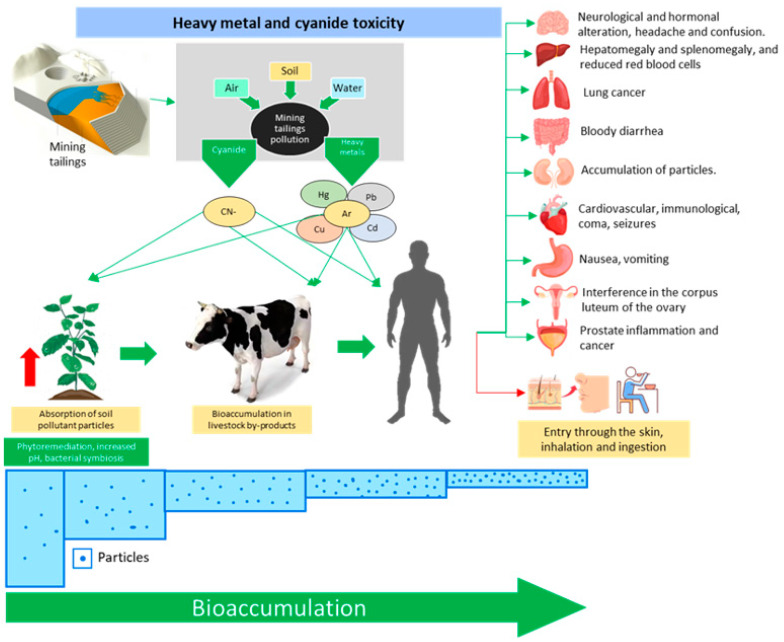
Diagram of socio-environmental risks linked to the chemical composition of mine tailings considering the presence of heavy metals and cyanide.

Heavy metals in food, such as vegetables, are another means of contamination for humans. In the case of cultivated foods, the uptake of metals by the roots is determined by the metal content in the soil, pH, type of soil and organic matter, cation exchange capacity, and the species and genotype of the plant. The ingestion of heavy metals by humans through the food chain can alter biochemical processes and induce toxicity in many organs of the body [126]. The immediate effects are a decrease in nutrients such as vitamin C and Fe, significantly affecting the immunity associated with malnutrition, while long-term effects are associated with increased cancer risks [127]. Likewise, the consumption of livestock products such as meat, milk, and eggs can be compromised when animals are fed with food from crops that have bioaccumulated heavy metals [128]. One of the foods most used in animal feed, corn, has high levels of heavy metals when planted near mine tailings [128]. Figure 24 shows a conceptual diagram that summarizes all aspects related to the toxicological process and socio-environmental risks discussed in this chapter.

### 4.2. Toxicity Issues Related to Cyanide

Cyanide is a highly toxic chemical compound that can be present in numerous solid, liquid, and gaseous forms at mining sites [67]. Solutions for the mining process are kept alkaline (typically above a pH of about 9.5) to prevent the formation of deadly hydrocyanic acid gas [63]. Exposure at high doses damages the brain and heart, and can result in coma, seizures, and death [70]. Exposure at low doses can lead to respiratory problems, heart pain, nausea, vomiting, headache, dizziness, mild confusion, abdominal cramps, and enlargement of the thyroid gland. A rice-grain sized dose of cyanide—ranging from 50 to 200 milligrams—can be fatal to humans [67].

Although cyanide forms in tailings storage facilities are partially broken down by direct sunlight, these processes can take months depending on weather conditions [129]. In addition, the original cyanide breaks down into numerous related compounds (for example, metal–cyanide complexes, cyanogens, thiocyanate, and cyanate) which, while less toxic than the original free cyanide, often remain toxic to fish, mammals, vegetation, and other forms of life [48,130].

Unfortunately, most of these other forms of cyanide mentioned above are not detected in routine laboratory tests. Standard metal mine surveys determine only the basic forms of cyanide, for example, Weak Acid Dissociable Cyanide (WAD) or total cyanide, and leave out the other forms. Therefore, many questions about the presence, persistence, and toxicity of cyanide and its related compounds remain unanswered [67].

Many modern gold mining facilities build and operate cyanide destruction plants; however, these fail to fully break down all toxic forms of cyanide and may even add concentrations of metals that can be toxic to fish. For example, rainbow trout (*Oncorhynchus mykiss*) tend to withstand toxicity, as they often significantly bioaccumulate cyanide in the organs, causing increased thyroid gland size, decreased hepatosomatic and splenosomatic indices, and reduced red blood cells. In this same context, there are many plants, mainly aquatic, that have the capacity to bioaccumulate cyanide from mine tailings. These have been used in the phytoremediation processes of both soil and water through the mechanism of enzymatic detoxification in symbiosis with microorganisms that have the ability to take in cyanide as a source of carbon and nitrogen and transform it into ammonia and carbonate that can later be used by plants [129].

In the case of wildlife such as mammals, the effects of cyanide contamination of mine tailings are worrisome. In combination with mercury, it usually presents serious lesions, mainly in the tissue of the kidney organs, although lesions have been found in the liver, blood, and spleen as well [130]. Wildlife tends to adapt very well to prolonged periods of exposure, eventually finding feeding mechanisms that improve their immunity [70].

**Figure 25:**
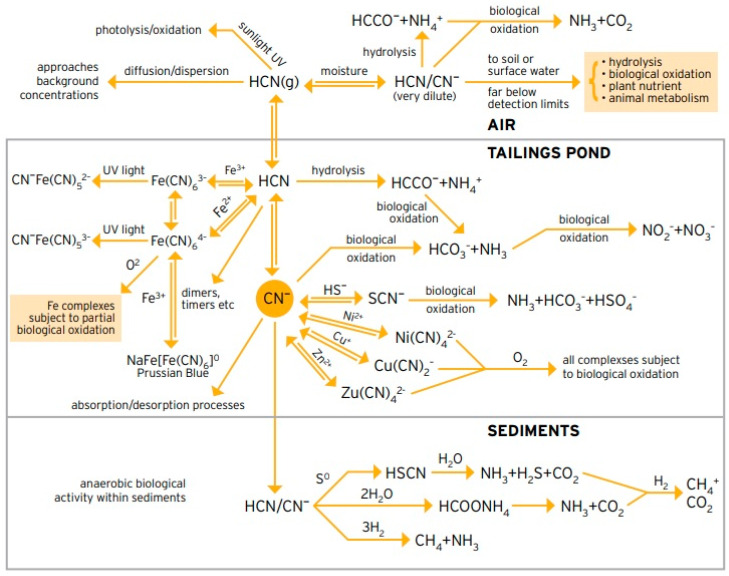
Cyanide chemical loss pathways in the environment [131].

Figure 25 shows a conceptual diagram that summarizes all aspects related to the cyanide chemical loss pathways in the environment discussed in this chapter.

**Figure 26:**
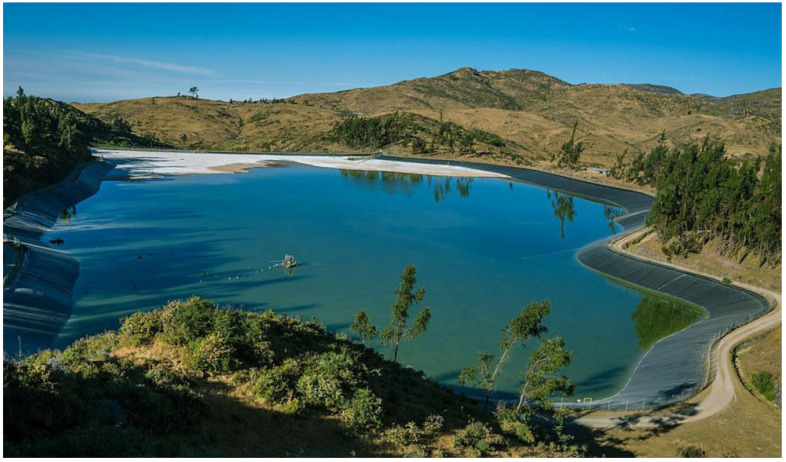
Gold tailings storage facility where the entire reservoir area is lined with a multilayer geosynthetics system.

Cyanide metal complexes eventually find their way to tailings storage facilities and then, potentially, into the environment. Cyanide will be lost in the tailings storage facilities and the environment through natural degradation reactions so that, in the long term, only the less toxic and strongly complexed forms remain [131]. Today, tailings storage facilities are designed to provide secure, long-term storage of materials containing such complexes and to avoid potential losses via seepage, overtopping, breaching, and piping failure. For example, Figure 26 shows a gold tailings storage facility where the entire reservoir area is lined with a multilayer geosynthetics system, which is made up of non-woven geotextiles, geosynthetic clay liner (GCL) and HDPE geomembrane with a thickness equivalent to 2 mm. The more toxic forms of cyanide in tailings storage facilities are measured as weak acid dissociable (WAD) cyanide, free cyanide and complexed forms [131].

## 5. Discussion

As a consequence of tailings storage facility failures around the world, the international community, institutions, and global groups such as the ICMM (International Council on Mining and Metals), the UN (United Nations) environmental program, and PRI (Principles for Responsible Investments) have developed Global Industry Standard on Tailings Management (GISTM), launched in August 2020, to regulate the operation of tailings storage facilities throughout their entire life cycle, including closure and post-closure (perpetuity), with the goal of zero damage to people and the environment and zero tolerance for human deaths [27,132]. Unfortunately, key aspects related to the management of mine tailings have not been considered in detail, including (i) the geochemical characterization of the mine tailings, (ii) the definition of process water, (iii) the generation of acid rock drainage, (iv) the potential for metal leaching, and (v) toxicity issues. There is a lack of definition and discussion of these issues in the Global Industry Standard on Tailings Management (GISTM). It is urgent to carry out comprehensive, multidisciplinary, trans-disciplinary, and holistic governance of mine tailings that incorporates these aforementioned issues into the Global Standard of Tailings Management for the Mining Industry in order to promote sustainable and responsible mining.

Environmental and geochemical issues must be integrated with geotechnical and hydraulic aspects related to the management of mine tailings. Due to the oxidation and leaching potential of metals in mine tailings, their physical and mechanical properties can be altered, reducing their resistance over time, which has a direct impact on the physical stability parameters of tailings dams built with cycloned tailings sands from total mine tailings (for example, see [133]). Geotechnical parameters such as cohesion and internal friction angle can suffer reductions during the useful life of a tailings storage facility, either due to the increase in confining pressures or to the complex and highly dynamic biogeochemical behavior of the mine tailings materials [95,134].

Mining companies must submit an environmental impact study (EIA) form well in advance of the intended use of chemical reagents in mine tailings generation processes, and the form must include a full description of the metallurgical process and chemical reagents applied in the mining project, including detailed information on the composition of the products and the CAS number. Data on toxicity, biodegradability, and bioaccumulation potential should be provided and, if such information does not exist, data on the ingredients of the chemical reagent should be available. The form must contain information on the proposed amounts of the chemical to be used along with details of its chemical and physical properties and its fate, including how the chemical will be managed and handled after use and its concentrations and amounts in mine tailings, process water, and drainage water. In general, it is recommended that all activities involving the use of chemical reagents in the extraction of minerals must comply with the best available techniques (BATs), best applicable practices (BAPs), and best environmental practices (BEPs) [135].

On the other hand, one of the best ways to reduce the toxicity of sulfide mineral tailings is desulfurization, that is, the removal of pyrite and potentially other sulfide minerals that are not removed in the normal process of floatation [6,8]. Desulfurized/depyritized tailings reduce acid generation and the potential for contaminant leaching, including the likelihood of leaching of arsenic, cadmium, and other toxic metals associated with sulfide ores. Desulfurized mine tailings can be deposited separately in a safer way, and can be used more sustainably as dam covers or traded as products [3].

In certain cases, mining companies may experience difficulties in meeting water quality standards due to the presence of metals such as copper, zinc, and/or cyanide in the supernatant process water pond from the mine tailings. Additionally, problems may be experienced downstream of the tailings storage facility due to the presence of foam and odor. In these cases, a series of measures must be implemented at both the tailings storage facility and the concentrator plant operation to achieve compliance and improve water quality downstream of the tailings storage facility. These measures can include:

- Installation of aeration devices to volatilize excess flotation reagents, reducing odor downstream of the tailings storage facility;
- Installation of an antifoam system;
- Installation of a sulfuric acid pH reduction system;
- Installation of a cyanide destruction series made up of a hydrogen peroxide system;
- Review and reduction of the use of chemical grinding reagents, and use of reagents that minimize the effects downstream of the tailings storage facility;
- Review and improvement of the water monitoring program to include visual observations for the presence of foam and odor;
- Development of a full site-wide water balance to enable better management and prediction of water quantity and quality.

**Figure 27:**
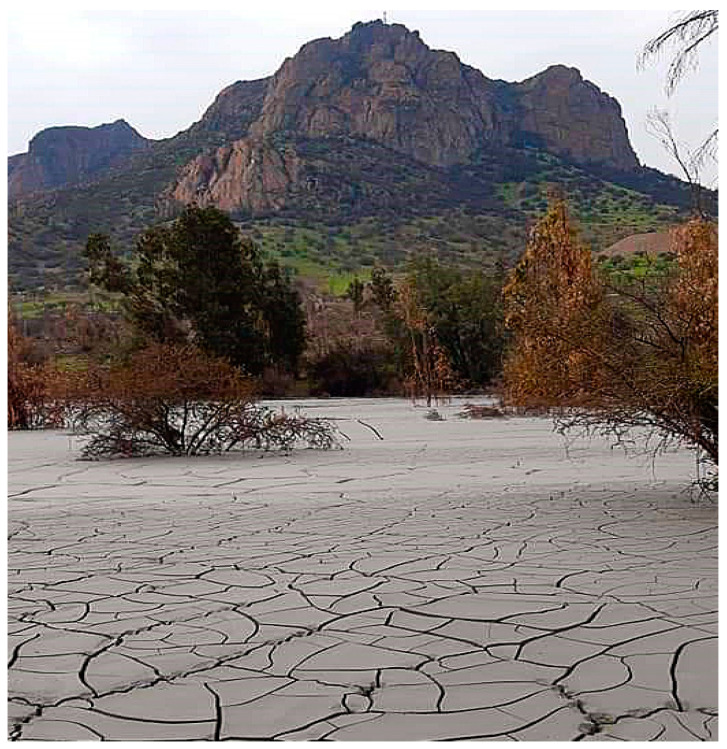
Soil, trees, and plants covered by mine tailings.

Mine tailings can generate many socio-environmental impacts, as is the case in both Chile and Peru. To store mine tailings, mountain valleys are used as storage sites. At such sites, it is only necessary to build a dam, which implies progressive filling of mine tailings in the area of the reservoir, eventually covering a large surface area that reaches hundreds of hectares in many cases [2,11]. This results in many species of local flora and fauna being sacrificed and remaining buried by mine tailings, such as plants, trees, vertebrate or invertebrate animals such as insects, birds, rodents, and others (Figure 27). A rescue and relocation plan for flora and fauna species is not usually carried out in the area that will be the reservoir of the tailings storage facility. We believe that it is important to incorporate these activities into the operating plans of mining companies with an ecological and sustainable purpose. In addition, in certain cases houses from old towns can be buried by millions of tons of mine tailings. There is a record of experiences where parts of the historical archaeological cultural heritage run the risk of being buried by mine tailings if adequate study and archaeological recovery is not carried out prior to the deposition of mine tailings. Human remains, utensils, ceramics, and textiles can be lost forever without being rescued for conservation if such measures are not taken.

In particular, the handling of gold mine tailings must be managed in a careful and controlled manner, mainly due to the cyanide residues present in the mine tailings and the potentially elevated levels of sulphates present in the supernatant process waters at tailings storage facilities [63]. The best available technologies (BATs) must be applied to contain the mine tailings and process water in a stable and controlled manner in gold mine tailings storage facilities. Such technologies include: (i) use of geosynthetic lining systems; (ii) dewatering of mine tailings through thickening and filtration; and (iii) detoxification processes of cyanide residues present in the mine tailings [48,67]. Is important to note that, in light of field experience in mining projects, the presence of dissolved cyanide, even at low concentrations, increases mercury mobility in groundwater. If necessary, reverse osmosis (RO) treatment plants should be implemented to reduce sulfate levels in supernatant process waters in order to meet local water quality standards.

Practical experience has shown that all types of mine tailings, whether conventional, thickened, paste, or filtered, release water over time and produce seepage that can alter the chemical quality of groundwater and surface water in river basins [12,16]. For this reason, it is extremely important that seepage control and collection systems be implemented in all tailings storage facilities, consisting of (i) lining systems with geosynthetics, (ii) cut-off trenches, (iii) plastic concrete slurry walls, (iv) grout–curtain systems, (v) basal drainage, (vi) seepage collection ponds/polishing ponds, and (vii) pumping wells and seepage monitoring [13,27].

Another aspect that must be controlled in the management of mine tailings is the emission of particulate material. After the mine tailings settle, consolidate, and dry out due to climatic action, a large part of the process water migrates either by evaporation or seepage, leaving the material mine tailings as no longer as a slurry but as a solid material susceptible to wind action [106]. Practical experience in both cycloned tailings sand dams and mine tailings storage reservoir areas has shown that if particulate material emission control measures are not applied the possibility of wind migration of particles with potential contaminants is high [7,107]. For this reason, the following dust generation mitigation alternatives must be implemented: (i) coverage of mine tailings with layers of soil or top soil; (i) implementation of phytostabilization with the use of native flora species; (iii) application of chemical binding agents; and (iv) dust retainers, among others [13].

The protection of native wildlife must be a priority as well; perimeter access to the tailings storage facilities must be closed, and domestic animals such as dogs, horses, cattle, sheep, and camelids, among others, must not be able to enter to drink water in the tailings storage facility. If possible, bird deterrents should be applied in order to prevent these animals from coming into contact with mine tailings and process water.

Finally, when carrying out technical and economic analyses of alternative ways of disposing of mine tailings, whether on land or in the sea, it is necessary to take into account the lessons learned from the past in many parts of the world, where an analysis was not carried out. Improper management of mine tailings that were deposited in the sea, generating socio-environmental impacts on marine and terrestrial ecosystems that persist to this day, include, for example, the cases of (i) Bahía de Chañaral, Chile, (ii) Bahía de Ite, Peru, and (iii) Bahía de Portman, Spain, among others [10,32,36,136,137,138,139,140]. Considering the lack of information and practical experience on the future effects of implementing the alternative of depositing mine tailings in the sea, it is necessary to apply the precautionary principle and act ethically and responsibly with respect to society and ecosystems. In this way, society can avoid repeating the mistakes of the past in present and future tailings management operations [141].

## 6. Conclusions

This article, being an independent study, seeks to promote knowledge and education of the community in general about what mine tailings are and their socio-environmental implications from a comprehensive, holistic, and multidisciplinary perspective. It is clear that today’s society requires mining activity for its development and daily living, just as mining requires society as a whole for its sustainability.

Operating and maintaining tailings storage facilities to meet global best practices for safety throughout their life cycle is a top priority for the mineral resource sector. Whether in operation or in a state of safe closure, it is important to continually review tailing facilities and procedures and to maintain the highest safety standard.

Due to their geochemical nature, mine tailings are materials that experience complex, highly variable, and dynamic spatiotemporal behavior within the environment, necessitating the consideration of the aspects of topography, climatology, geography, hydrology, hydrogeology, and edaphology, among others. The potential risk of generating acid rock drainage (ARD) in mine tailings due to biogeochemical reactions of oxidation, hydrolysis, and leaching of metals incurs a danger to both the ecosystem and to humans, and must be monitored and controlled at all lifetimes in a tailings storage facility.

Water that has contacted mine tailings, known as supernatant process water, can contain high concentrations of: (i) non-metallic compounds such as sulfates, nitrates, chlorides, and ammonia; (ii) high concentrations of metals and dissolved metalloids such as: As, Cr, Cd, Fe, Cn, Pb, Mn, Mo, Cu, and rare earths elements (REEs), among others; and (iii) traces of metallurgical reagents (xanthates, pine oil, sodium cyanide, sodium sulphate, and copper, among others. As such, this water must be managed in a controlled manner within the mining process in a closed circuit, promoting its reuse, mitigating leaks, and avoiding leaks or discharges without prior treatment into the environment that alter the chemical quality of surface and groundwater of the basins.

A metallurgical process plant for the treatment of gold ore and production of gold mine tailings in terms of cyanide treatment must be designed taking into account the requirements of the International Cyanide Management Code, which contemplates the highest world standard in the management of this substance.

In addition, because the interactions of physical, biological, chemical, toxicological, engineering, and socioeconomic factors with human activities are extremely complex, the general historical trend has been to simply present the results of environmental impact assessment studies (EIA) of mine tailings management projects in isolation rather than fully integrating them through interdisciplinary, transdisciplinary, and multiscalar (spatial and temporal) analysis, thereby obviating the study of the characteristics of a complex system. For this reason, the participation of multidisciplinary teams in a transdisciplinary approach is necessary in the design, operation, and closure of tailings storage facilities, which should involve carrying out an interdisciplinary and transdisciplinary study considering the complexity of tailings management beyond conducting just engineering studies.

According to the contents presented in this article, mine tailings can be highly toxic materials for both ecosystems and humans; for this reason, mine tailings cannot be considered inert and innocuous materials, and must be handled carefully in a responsible and controlled manner to avoid socio-environmental impacts due to potential spills, leaks, or collapses of tailings storage facilities.

Today, mine tailings are no longer seen as mining waste but as a georesource, and it is expected that in the near future these materials will be reprocessed as a new way of mining in order to recover valuable metals, remnants, and other new chemical elements of technological interest, such as Cobalt, Vanadium, Gallium, Germanium, and rare earth elements (REEs). These elements are used in the development and manufacture of electronic items, materials for renewable energy generation systems, and aerospace materials, among others. This will mean carrying out responsible management of mine tailings with new metallurgical technologies for mineral processing while applying the principles of circular economy and sustainability, representing a new opportunity for the mining industry to invest in redeposition and responsible closure of mine tailings storage facilities while reducing potentially dangerous and toxic elements in the environment.

To promote the reduction of socio-environmental risks considering the chemical composition of mine tailings, it is necessary to implement new approaches focused on sustainability, such as applying the principles of reduce, reuse, and recycling to the management of mine tailings. New metallurgical techniques for the benefit of minerals must be applied to reduce the amount of mine tailings; in addition, a way to reuse mine tailings as construction materials for roads or houses must be sought, and the reprocessing of mine tailings must be promoted to obtain other beneficial metals necessary for new emerging technologies in the Industry 4.0 context. New uses for mine tailings must be explored, breaking the contemporary status quo and seeking innovative alternatives focused on green mining.

Finally, it should be noted that the mining industry, in seeking constant improvement and in search of sustainable development, must implement a high-level culture of safety and responsibility. In addition, it will require agreements and the joint support of companies, communities, academia, governments, NGOs, and citizens in general to obtain the social license to operate future tailings storage facility projects. It is necessary for the governance of mine tailings to implement the highest standards of design, construction, operation, closure and post-closure stages of tailings storage facilities in order to guarantee the safety of people reduce the environmental footprint of mining activities. This implies making significant efforts, unifying criteria, and creating political will in the governance of mine tailings, with the objective of implementing (i) the best available technologies (BATs), (ii) the best applicable practices (BAPs), (iii) the best environmental practices (BEPs), (iv) the respective capital investments that will translate into Corporate Social Responsibility (CSR), (v) improvement around issues related to Environmental, Social, and Governance (ESG) issues, (vi) support for the achievement of the sustainable development goals (SDGs), and (vii) a sustainable culture of care for the environment and trust in the actions of the mining industry on the part of societal stakeholders.

## Figures and Tables

**Table 1:** Typical metallurgical reagents present in copper mine tailings.

Classification	Reagents Name	Quantity Used (g/ton of Tailings)
Acids	Sulfuric Acid (H_2_SO_4_)	500–2500
Alkalis or pH Regulators	Lime	500–3000
	Sodium carbonate	550–3500
	Sodium hydroxide	200–800
Modifiers (depressants, activators and dispersants)	Copper sulfate	50–550
	Sodium cyanide	10–50
	Zinc sulfate	50–2000
	Sodium sulfate	50–3000
	Sodium silicate	50–3000
	Sulfur dioxide	50–850
	Starch	50–150
Collectors	X-Amylxanthate	30–300
	X-Isopropylxanthate	10–30
	X-Ethylxanthate	5–25
	Diesel oil	10–100
	Amine	30–300
	Aniline dicresyl dithiophosphate plus thiocarbonilide	10–75
Frothers	Polypropylene glycol methyl ether	10–25
	Hexylic acid	10–50
	Pine oil	5–25
	HBTA frother	25–100
	Carbon	5–30
Flocculants	Floeger 913-SH	10–25
	Floeger 923-SH	10–25
	Tec-2050	10–25
	Magnafloc 1011	10–25
	Magnafloc 155	10–25
	Magnafloc 2025	10–25
	Magnafloc 333	10–25
	Rheomax 1050	10–25
	Orifloc AP 2020	10–25
	Superfloc A-110	10–25

**Table 2:** Typical chemical elements (metals and heavy metals) present in copper mine tailings.

Chemical Symbol	Chemical Element Name
Cu	Copper
Al	Aluminum
Cd	Cadmium
Pb	Lead
Fe	Iron
Cr	Chromium
Co	Cobalt
Mn	Manganese
Mo	Molybdenum
Ni	Nickel
V	Vanadium
Zn	Zinc
U	Uranium
REEs	Rare Earth Elements

**Table 3:** Typical chemical elements (metalloids) present in copper mine tailings.

Chemical Symbol	Chemical Element Name
Sb	Antimony
As	Arsenic
B	Boron
Si	Silicon
Ge	Germanium
Te	Tellurium
Po	Polonium

**Table 4:** Typical chemical elements (non-metals) present in copper mine tailings.

Chemical Symbol	Chemical Element Name
Cl^−^	Chlorides
SO_4_	Sulfates
NO_3_	Nitrates
NH_3_	Ammonia

**Table 5:** Typical chemical elements (metals and heavy metals) present in gold mine tailings.

Chemical Symbol	Chemical Element Name
Au	Gold
Ag	Silver
Hg	Mercury
Ca	Calcium
CN	Cyanides

**Table 6:** Maximum Permissible Limits (LMP) and Environmental Quality Standards (ECA) considered for spillage of copper and gold mining tailings in Peru, Chile, and internationally [7].

Total Parameters	Peru		Chile		International LMP ^(3)^
	LMP	ECA ^(1)^	LMP	ECA	
Water for irrigation (mg/L)					
*Arsenic*	0.08	0.05	0.1	0.1	0.1
*Cadmium*	0.04	0.005	0.01	0.01	0.01
*Cyanide*	0.8	0.1	0.2	0.1	--
*Copper*	0.4	0.2	0.2	0.2	0.017
*Chromium*	0.08	0.1	0.1	--	0.55
*Iron (dissolved)*	1.6	1	5	0.3	0.5
*Manganese*	--	0.2	0.2	0.01	--
*Mercury*	0.0016	0.001	0.001	0.001	--
*Lead*	0.16	0.05	5	5	0.065
*Zinc*	1.2	2	2	0.2	0.2
*pH*	6–9	6.5–8.5	5.5–9	6.5–8.5	--
Agricultural land (mg/Kg)		^(2)^			
*Arsenic*	--	50			20
*Barium*	--	750			--
*Cadmium*	--	1.4			3
*Chromium*	--	0.4			100
*Lead*	--	70			100
*Free Cyanide*	--	0.9			2 ***
*Mercury*	--	6.60			0.50 **
*Cyanide*	--	0.09			300

^(1)^ Decreto Supremo N° 010-2010-MINAM, Peru. ^(2)^ Decreto Supremo N° 011-2017-MINAM, Peru. ^(3)^ The international LMPs for water and soil were taken by the WHO and the FAO. ** Canada Quality Guide 2003 (PEL). *** U.S. EPA (Environmental Protection Agency) 2021.

## Data Availability

The data presented in this study are available on request from the corresponding author.

## Abbreviations

TSF	Tailings Storage Facility
BATs	Best Available Technologies
BAPs	Best Applicable Practices
BEPs	Best Environmental Practices
GISTM	Global Industry Standard on Tailings Management
ICMM	International Council on Mining and Metals
UN	United Nations
PRI	Principles for Responsible Investment
CTD	Conventional Tailings Disposal
TTD	Thickened Tailings Disposal
PTD	Paste Tailings Disposal
FTD	Filtered Tailings Disposal
ARD	Acid Rock Drainage
Cw	Slurry tailings solids content by weight
mtpd	Metric tonnes per day
CAS Number	Chemical Abstracts Service registry number
pH	Potential of Hydrogen index
CIL	Carbon-in-Leach
CIP	Carbon-in-Pulp
GCL	Geosynthetic clay liner
HDPE	High density polyethylene
INCO	Air cyanide destruction method with sulfur dioxide (SO_2_)
WAD	Weak Acid Dissociable cyanide
CCD	Counter-Current Decantation
RO	Reverse Osmosis
CSR	Corporate Social Responsibility
ESG	Environmental Social and Governance
SDGs	Sustainable Development Goals
WHO	World Health Organization
EPA	Environmental Protection Agency
FAO	Food and Agriculture Organization of the United Nations
masl	Meters above sea level
